# Curcumin, Resveratrol and Berberine as Emerging Therapeutic Agents in Treatment of Gliomas: A Comprehensive Review

**DOI:** 10.3390/molecules31213835

**Published:** 2026-10-23

**Authors:** Dominika Kunachowicz, Julia Kostrzewa, Paulina Tomecka, Anna Karwowska, Aleksandra Wojno, Agata Wojno, Oliwia Cichy, Milena Chmielewska, Paulina Lenkiewicz, Barbara Białynicka-Birula, Anna Choromańska

**Affiliations:** 1 Department of Pharmaceutical Biochemistry, Faculty of Pharmacy, Wroclaw Medical University, Borowska 211a, 50-556 Wroclaw, Poland; 2 The Students’ Research Group, SKN No. 148, Department of Molecular and Cellular Biology, Faculty of Pharmacy, Wroclaw Medical University, Borowska 211A, 50-556 Wroclaw, Poland; julia.kostrzewa@student.umw.edu.pl (J.K.); paulina.tomecka@student.umw.edu.pl (P.T.); oliwia.cichy@student.umw.edu.pl (O.C.); paulina.lenkiewicz@student.umw.edu.pl (P.L.); barbara.bialynicka-birula@student.umw.edu.pl (B.B.-B.); 3 Faculty of Medicine, Wroclaw Medical University, 50-367 Wroclaw, Poland; anna.karwowska@student.umw.edu.pl (A.K.); aleksandra.wojno@student.umw.edu.pl (A.W.); agata.wojno@student.umw.edu.pl (A.W.); milena.chmielewska@student.umw.edu.pl (M.C.); 4 Department of Molecular and Cellular Biology, Wroclaw Medical University, Borowska 211A, 50-556 Wroclaw, Poland

**Keywords:** curcumin, resveratrol, berberine, brain cancers, glioma, glioblastoma, delivery systems

## Abstract

Brain tumors represent a major clinical challenge due to their complex biology, inherent resistance to conventional therapies and the limited delivery of anticancer agents across the blood–brain barrier. Natural phytochemicals such as curcumin, resveratrol and berberine have gained increasing attention for their multi-targeted anticancer properties, including induction of apoptosis, inhibition of proliferation, suppression of mitochondrial and BCL-2-family signaling and modulation of the tumor microenvironment. This comprehensive review critically examines current evidence on the use of curcumin, resveratrol and berberine in the context of brain tumor treatment, with an emphasis on gliomas’ mechanisms of action, pharmacokinetic limitations, and potential for integration into novel delivery platforms. We discuss how these compounds interact with key pathways in brain tumors—including BCL-2 inhibition, PI3K/AKT/mTOR signaling and epigenetic regulation—and the emerging strategies to overcome glioma-specific obstacles such as blood–brain barrier permeability and microenvironmental hypoxia. Furthermore, the review explores innovative formulations and carrier systems that enhance brain uptake and bioavailability of these agents, bridging the gap between preclinical promise and clinical translation. By identifying current gaps and proposing future research directions, this article aims to provide a roadmap for harnessing curcumin, resveratrol and berberine as adjuvant or combinatorial treatments in brain tumor therapy, focusing particularly on glioma treatment. The adoption of such phytochemicals may open new avenues for improved outcomes in patients with highly refractory brain malignancies.

## 1. Introduction

**Figure 1:**
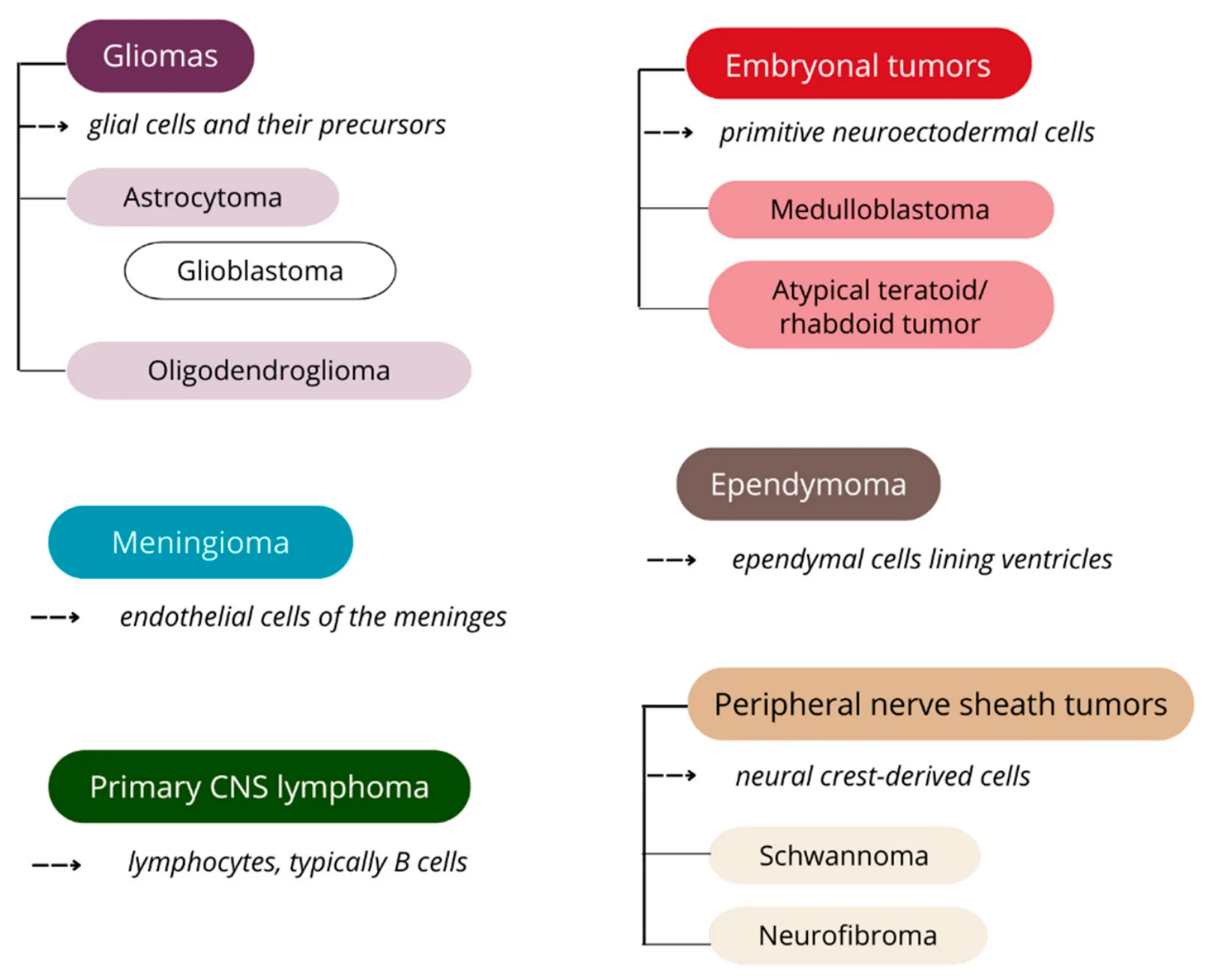
Classification of brain and central nervous system cancer types.

Brain and central nervous system (CNS) cancers, though relatively rare compared to many systemic malignancies, represent a substantial health burden due to their high mortality and limited therapeutic options. Primary malignant brain tumors occur in about 3.5 per 100,000 individuals annually and represent approximately 2% of cancer diagnoses, with substantial geographic variation in incidence [1]. Gliomas—particularly glioblastoma (GBM)—are known as the most prevalent and aggressive types of brain tumors; glioblastoma (formerly “glioblastoma multiforme”) alone accounts for the majority of malignant primary brain tumors in adults and carries a median survival of barely more than one year despite maximal surgical, radiotherapeutic, and chemotherapeutic intervention. Its frequency increases after the age of 40, with the highest incidence found in those aged 75 to 84 [2,3]. Lower-grade gliomas, which grow from glial cells or their precursors, primarily affect those younger than 50 and are categorized into astrocytomas and oligodendrogliomas. Another malignant brain tumor in adults is primary central nervous system lymphoma (PCNSL), a rare form of non-Hodgkin lymphoma, which occurs in the brain, eyes, or leptomeninges, with no proven extracranial placement [2]. A group of ependymal tumors, deriving from ependymal cells lining ventricles, is also distinguished. Ependymomas can develop in both children and adults within the supratentorial brain, posterior fossa, and spine [2]. The most frequent primary CNS benign tumor in adults is meningioma [2,4]—a neoplasm originating from meningothelial cells, residing in the meninges, which provide barrier function to the CNS [5]. The division of brain and central nervous system tumor types, along with their cells of origin, is shown in Figure 1.

A scope note applies to the rest of this review: although the tumor types introduced above span the full range of primary and secondary brain neoplasms, the preclinical and clinical evidence available for curcumin, resveratrol, and berberine—reviewed in Section 3, Section 4 and Section 5—relates almost exclusively to glioma and glioblastoma models. We are not aware of comparable evidence in meningioma, medulloblastoma, ependymoma, primary CNS lymphoma, or brain metastases for these three compounds, and this evidence gap should be understood as a limitation of the underlying literature rather than of this review’s coverage of it.

Depending on cancer type, location and stage, between 20% and 45% of cancer patients will develop brain metastases (BMs) during their lifetime. Such secondary tumors, formed by malignant cells that have spread to the brain from elsewhere in the body, occur more frequently than primary brain tumors in both adults and children. The presence of intracranial metastases is associated with a grim prognosis (in such cases, mean survival does not exceed a year), notable neurodegeneration, and a decline in overall quality of life [6]. Most BMs originate from lung, breast, melanoma, renal cell, and colorectal cancers [7].

GBM and BMs are the most prevalent and challenging malignant brain tumors to treat. Achieving the complete removal of a GBM tumor is particularly tough due to its invasive cells that spread into nearby brain tissue. Patients with GBM face an unfavorable prognosis, with a median survival of about 15 months, a five-year survival rate below 10%, and a recurrence rate around 90%. Unfortunately, advancements in treating brain tumors have been minimal over the past decade. Treating brain tumors is challenging due to significant variability between patients, tumor heterogeneity, and resistance development, frequently observed for brain cancers [8,9]. The presence of the blood–brain barrier (BBB), established by the endothelial cells of brain microvessels that line the cerebral capillaries extending into the brain and spinal cord, is another limiting factor in the context of treating intracranial tumors—although essential for proper neuronal function and protection [10,11], it acts as an obstacle in delivering therapeutics to the brain parenchyma. This enables malignant tumors to reside within the brain, evading therapeutic measures and resulting in increased patient mortality [6]. All of these underscore the need to develop new therapeutic strategies that go beyond conventional approaches, including the use of natural substances in the fight against cancer.

Natural compounds play dual roles in cancer. Some of them, as pro-oxidants, induce oxidative stress in cancer cells, promoting cell death, which enhances standard therapies. In contrast, their antioxidant function protects normal cells, potentially preventing tumor recurrence and reducing cancer risk. This dual action makes them useful both as therapeutic agents and in cancer prevention [12]. Exploration of plant-derived natural compounds in brain tumor therapy has become an important research focus, primarily due to the limitations of conventional therapies. Traditional approaches like chemotherapy and radiation, though central to current treatment strategies, often result in high toxicity levels and severe side effects, affecting healthy brain tissues [13]. Studies on numerous plant-derived agents reveal neuroprotective effects that make these compounds ideal candidates for brain cancer therapies, as they can modulate complex cellular responses without the same level of toxicity associated with synthetic drugs [14]. Furthermore, many natural compounds are known for their ability to cross the blood–brain barrier through both transcellular and paracellular diffusion [15]. In recent studies, compounds like ferulic acid and curcumin have demonstrated potential in mitigating tumor growth and enhancing immune responses [16]. By activating multiple signaling pathways, these compounds induce apoptosis, inhibit angiogenesis, a process crucial for tumor development, and modulate the body’s immune response, offering a holistic approach to combating cancer progression [17]. Such multi-targeted interventions are essential in addressing the complexity and adaptive nature of brain tumors, making natural compounds a valuable area for future drug development [18].

Research into natural compounds for brain tumor treatment has significantly progressed, reflecting the need for therapies with enhanced efficacy and reduced toxicity. A central focus has been on compounds with multi-target mechanisms that can modulate cellular pathways crucial to cancer development, such as inflammation, apoptosis, and angiogenesis. For instance, curcumin—a polyphenolic compound found in turmeric—has shown the ability to disrupt cancer cell growth and modulate signaling pathways, especially in glioblastoma models. Its ability to enhance the effect of other drugs when combined further supports its therapeutic value in brain tumor treatment [19]. Berberine exhibits pro-apoptotic activity and represents a promising candidate for combined cancer therapy. Its low toxicity toward normal cells underscores its clinical safety and therapeutic potential in biochemical disorders [20]. Resveratrol reduces the risk of multidrug resistance (MDR) by targeting multiple cellular pathways involved in carcinogenesis and chemo-/radioresistance, thereby enhancing its combined effect with chemotherapeutic agents [21]. Herein, we will review and discuss recent findings regarding the potential of three important bioactive compounds: resveratrol, curcumin, and berberine, in stand-alone or supportive treatment of brain tumors, in both preclinical studies and clinical settings.

Curcumin and resveratrol are both polyphenols, whereas berberine is a benzylisoquinoline alkaloid with a fundamentally different scaffold, physicochemical profile, and pharmacokinetic behavior (notably, its permanently charged quaternary nitrogen, discussed in Section 3.3). These three compounds were selected because, despite their structural diversity, they converge on a shared and partially overlapping set of oncogenic pathways in glioma models (PI3K/Akt/mTOR, NF-κB, STAT3, and apoptosis/autophagy regulation), while each also offers a distinct, complementary angle: curcumin as the most extensively reformulated compound in clinical-stage delivery research, resveratrol as the compound with the most detailed human pharmacokinetic characterization, and berberine as a structurally distinct alkaloid with an established, independent safety record from its long-standing metabolic indications. This combination of mechanistic overlap and pharmacological contrast was, in our view, more informative for a comparative review than a single-class focus. Other frequently studied natural compounds with reported anti-glioma activity, such as quercetin, epigallocatechin gallate (EGCG), and sulforaphane, were not included; this was a pragmatic scoping decision to keep the review to a manageable and coherent comparison set, not a judgment that these compounds are less promising, and we see a systematic comparison across this broader class of dietary phytochemicals as a natural direction for future work.

### 1.1. Literature Search Strategy

This review was conducted as a narrative, critically appraised synthesis rather than a systematic review; no formal protocol was registered. Literature was identified through targeted searches of PubMed/MEDLINE and Scopus conducted between June and September 2026. Publications from 2007 to 2026 were considered, with a major focus on the reports published in the last 10 years (2016–2026) while retaining earlier studies when they provided important mechanistic or historical context. Searches combined the compound names (“curcumin”, “resveratrol”, “berberine”) with brain-tumor-related terms (“glioma”, “glioblastoma”, “brain tumor”, “CNS neoplasm”, “brain cancer”) and mechanism/translational terms (“apoptosis”, “blood-brain barrier”, “bioavailability”, “nanoformulation”, “clinical trial”), supplemented by manually searching the reference lists of retrieved articles and recent reviews, and by searching ClinicalTrials.gov and the WHO ICTRP for trial records. Clinical trial registries were searched on 6 October 2026, using ClinicalTrials.gov and the WHO International Clinical Trials Registry Platform (ICTRP). Search terms included the individual compound names (“curcumin”, “resveratrol”, and “berberine”) and disease-related terms (“glioma”, “glioblastoma”, and “brain tumor”). Where applicable, compound names were combined with disease-related terms to identify trial records relevant to the use of these agents in glioma. Records were screened for relevance to the therapeutic application of the compounds in glioma and for information on clinical investigation, trial status, and translational development. We emphasize that this remains a narrative review: study selection reflects the authors’ judgment of relevance and was not performed independently in duplicate, and no formal risk-of-bias or evidence-quality assessment (e.g., SYRCLE for animal studies) was applied to the individual primary studies discussed below.

### 1.2. Positioning Relative to Existing Reviews

Several single-compound reviews already cover curcumin or resveratrol in brain tumors in detail, including some cited in this manuscript [22,23,24,25,26,27], and we are aware of directly overlapping reviews published during the preparation of this manuscript, including a 2025 review of curcumin in glioblastoma [28] and a 2026 review of curcumin and nanocurcumin in glioblastoma [29], as well as reviews specifically addressing resveratrol in glioma (e.g., [30,31]), a combined curcumin/resveratrol review [32], and a broader review of curcumin in brain diseases generally rather than brain tumors specifically [33]. To our knowledge, none of these existing reviews jointly cover curcumin, resveratrol, and berberine, and none systematically contrast the three compounds’ in vitro effective concentrations. The addition of berberine—structurally and pharmacokinetically distinct from the two polyphenols—is intended to let this review draw out shared and divergent translational obstacles across a structurally diverse compound set, rather than to catalog three compounds for breadth alone. Where our conclusions overlap with these parallel reviews (in particular regarding poor bioavailability and the current scarcity of controlled clinical trials), we consider this convergence rather than redundancy, as it was reached largely independently and from a comparative, multi-compound perspective.

## 2. Pathophysiology of Brain Tumors

### 2.1. Mechanisms of Brain Tumor Development

The development of tumors is a complex process driven by various genetic and molecular alterations, primarily involving genetic mutations, oncogenes, and tumor suppressor genes. These elements interact and lead to uncontrolled cell growth, resistance to apoptosis, and metastatic capabilities. In general, according to the widely accepted hypothesis, genetic mutations are the fundamental drivers of cancer development. They can occur due to environmental factors, inherited susceptibilities, or errors during DNA replication. These mutations alter normal cellular functions by disrupting regulatory mechanisms that control cell proliferation and survival [34]. Specific types of mutations, such as point mutations, deletions, and amplifications, can significantly affect gene function, leading to either gain-of-function or loss-of-function outcomes. Oncogenes are mutated or overexpressed forms of proto-oncogenes, which are genes that, in their normal state, regulate cell growth and differentiation. When proto-oncogenes mutate, they become permanently activated, resulting in continuous cell division even in the absence of growth signals. For instance, mutations in the RAS gene family—common in many cancers—promote signaling pathways that drive uncontrolled cellular proliferation [35]. Such mutations essentially remove the brakes on cell division, allowing for rapid tumor growth. Tumor suppressor genes, in contrast to oncogenes, act as the cellular defense mechanism against tumor development by inhibiting cell proliferation, repairing DNA damage, and initiating apoptosis when necessary. The loss of function in tumor suppressor genes—often through genetic mutations—results in the loss of these protective roles, contributing to cancer progression. The TP53 gene, commonly known as the “guardian of the genome,” is one of the most frequently mutated tumor suppressor genes in cancer [36]. When TP53 is inactivated, cells fail to repair damaged DNA and avoid programmed cell death, creating an environment conducive to further mutations and tumor evolution. The cumulative effect of genetic mutations, activation of oncogenes, and inactivation of tumor suppressor genes drives tumor development. Over time, these alterations accumulate, leading to a progressively more malignant phenotype that includes increased invasiveness, resistance to treatment, and metastatic potential [37]. This multistep process is reflected in the “multi-hit” hypothesis, where multiple genetic events must occur before a normal cell transforms into a cancerous one [38].

Brain tumors are characterized by distinct genetic and molecular alterations, rarely observed in non-CNS cancers. One of the most defining molecular features of diffuse gliomas, found in approximately 80% of WHO grade II/III glioma and 70% of secondary GBM cases, is mutation of the isocitrate dehydrogenase genes IDH1 and IDH2. Mutations of certain arginine residues lead to the production of D-2-hydroxyglutarate, which depletes the Krebs cycle, inducing substantial reprogramming of numerous metabolic processes [39]. Primary GBM often (50–60% of cases) exhibits amplification of the EGFR gene, including the EGFRvIII variant. This results in persistent activation of downstream pathways such as PI3K-Akt-mTOR and RAF-MEK-ERK, mediated by receptor tyrosine kinase activity of EGFR, and influencing proliferation, survival and metabolism [40]. On the other hand, an important genetic hallmark of oligodendrogliomas is the loss of the short arm of chromosome 1 and the long arm of chromosome 19, known as 1p/19q-codeletion. Interestingly, it is a good prognostic factor, related to the high success rate of treatment in tumors bearing this mutation [41]. Another event that can be considered beneficial in treating brain tumors is promoter methylation of MGMT (O-6-methylguanine-DNA methyltransferase), a crucial enzyme of DNA repair. Reduced transcription and translation of MGMT, which is a consequence of such methylation, is associated with a lower capacity to fix DNA lesions and therefore predicts sensitivity to alkylating agents such as temozolomide [42]. Conversely, specific cytosine–thymine transitions in the telomerase reverse transcriptase (TERT) gene promoter, frequently observed in glioblastomas, gliomas, and certain meningiomas, enable unhindered proliferation due to restored telomerase activity, correlating with high invasiveness and poor prognosis [43,44].

A terminological note applies throughout this review: many of the preclinical studies discussed below predate the 2021 WHO classification and describe their models using older nomenclature (e.g., “stage” rather than “grade,” or “glioblastoma multiforme” without reference to IDH status). Under the current classification, “glioblastoma” is, by definition, an IDH-wildtype, WHO grade 4 tumor, whereas IDH-mutant astrocytic tumors are now classified separately (WHO grade 2–4 astrocytoma, IDH-mutant). Because IDH status was not systematically reported in most of the older cell-line and xenograft studies cited here, we have preserved the terminology used by the original authors where the IDH status of the model cannot be inferred with confidence, and we flag this as a limitation of the underlying evidence base rather than of the present review.

Most solid cancers derive from epithelial tissue (carcinomas), while the vast majority of primary brain cancers such as gliomas, astrocytomas, or oligodendrogliomas arise from glial or neural precursor cells. Therefore, these cancers exhibit several unique pathophysiological features compared to the tumors located in other parts of the human body. Apart from oncogenic mutations and epigenetic alterations, neuronal activity is also a factor that, through regulating precursor cell proliferation, differentiation and function, contributes to the progression of brain tumors. In the case of gliomas, neuronal inputs are mediated through paracrine signaling and direct electrochemical communication through neuron-glioma synapses [45].

### 2.2. Challenges in Brain Tumors Treatment

Brain tumors present unique challenges in treatment because their pathophysiology differs fundamentally from that of other solid cancers. They originate from distinct neural and glial cell populations, whose biology, differentiation potential, and signaling pathways diverge significantly from epithelial- or mesenchymal-derived tumors found elsewhere in the body. Their growth patterns are also atypical: instead of forming well-demarcated masses, many brain cancers infiltrate surrounding neural tissue in a diffuse, finger-like manner; therefore, their complete surgical resection is usually unlikely without damaging essential brain structures, especially in high-grade cancers [46]. The location of tumors in or adjacent to areas controlling critical autonomic centers, motor function, speech, or vision often hinders aggressive therapeutic interventions, constraining radiotherapy dose and/or acceptable margin of nearby critical structures [47].

Moreover, the tumor microenvironment in the brain is profoundly different from that of peripheral tissues. It is shaped by specialized cell types such as astrocytes, microglia, oligodendrocytes, Treg cells or M2 phenotype macrophages, and unique extracellular matrix (ECM) components, all of which interact with tumor cells and influence their survival, invasiveness, and limit the efficacy of anticancer therapies [48]. Additionally, the immune landscape of the central nervous system is more immunosuppressive and tightly regulated, further limiting effective antitumor responses. Brain tumors are also characterized by distinct molecular alterations that drive their development and progression, including mutations in IDH1/2, methylation status of the MGMT promoter affecting chemoresistance, amplification or mutation of EGFR, and alterations in genes regulating chromatin remodeling. These genetic and epigenetic features underscore the biological specificity of brain cancers and contribute to their particularly aggressive course and therapeutic refractoriness [49,50]. Understanding these unique aspects is essential for designing effective treatment strategies tailored to the complex biology of brain tumors.

Apart from that, major challenges arise from the presence and function of the blood–brain barrier (BBB) and the intrinsic heterogeneity of tumor cells. BBB is a highly selective, protective barrier composed of endothelial cells that tightly regulate what substances can enter the brain. While this barrier is essential for protecting neural tissue from toxins and pathogens, it also restricts the passage of most chemotherapeutic agents, limiting their effectiveness in treating brain tumors. This is especially problematic in aggressive brain cancers like glioblastoma, where therapeutic drugs need to penetrate the BBB to reach tumor cells [51]. Various strategies, including nanoparticles and drug carriers, have been explored to enhance drug delivery across the BBB, but achieving effective concentrations within brain tumors remains a significant hurdle [52].

A critical consideration in translating the in vitro antitumor activity of curcumin, resveratrol, and berberine into potential glioma therapies is whether pharmacologically relevant concentrations can be achieved and sustained within the tumor after systemic administration. Evidence of blood–brain barrier (BBB) penetration should be interpreted in the context of the route of administration, formulation, dose, sampling time, and chemical species quantified. Plasma concentrations cannot be used as a direct surrogate for brain or tumor exposure, and measurements in whole-brain homogenates do not necessarily reflect concentrations in the brain parenchyma or glioma tissue. Furthermore, the detection of a parent compound or its metabolites in brain tissue does not, by itself, demonstrate that sufficient concentrations of the pharmacologically active species are available at the tumor site to reproduce the effects observed in vitro. For example, animal studies have demonstrated measurable brain exposure to curcumin and resveratrol, whereas berberine has generally been associated with limited systemic bioavailability and low brain concentrations. However, these findings derive from different experimental conditions and cannot be directly compared or extrapolated to human glioma treatment. Furthermore, tissue detection of a parent compound or its metabolites does not establish that pharmacologically active, unbound concentrations are achieved at the target site [53,54,55].

Although tumor-associated vascular abnormalities may increase permeability in some regions, other regions retain substantial BBB integrity. In addition, infiltrating glioma cells may extend beyond the contrast-enhancing tumor core into brain tissue with a relatively intact BBB, potentially limiting drug exposure at sites of residual disease. Consequently, drug accumulation in the tumor core or evidence of BBB penetration in healthy animals cannot be assumed to demonstrate adequate exposure throughout the tumor. The interpretation of pharmacokinetic evidence should therefore distinguish systemic exposure from brain and tumor distribution and, where data permit, compare measured tissue concentrations with concentrations associated with antitumor activity in relevant glioma models. In the absence of direct tumor pharmacokinetic data, the ability of these compounds to achieve therapeutically effective exposure in glioma should be regarded as unresolved rather than established [56,57].

Another related factor is the lack of a typical lymphatic system in brain tissue. An alternative, the so-called glymphatic system, composed mainly of cerebrospinal fluid and regulated by glial cells, is less efficient in the clearance of tumor-associated antigens, leading to lowered antigen presentation and fewer T cells being activated. Due to this poor immune infiltration, brain tumors are often referred to as “cold”. In addition, compared to extracranial cancers, primary brain tumors exhibit lower expression of immune checkpoint molecules and reduced T-cell infiltration, resulting in decreased success of immunotherapy [58].

Tumor heterogeneity refers to the diverse genetic and phenotypic profiles of cancer cells within a single tumor. This variability is particularly pronounced in brain cancers, where cells may exhibit different mutations, growth rates, and responses to treatment within the same tumor mass. As a result, therapies that target specific mutations or signaling pathways may only affect a subset of tumor cells, allowing resistant populations to survive and proliferate [59]. Additionally, brain tumors often contain stem-like cells that contribute to recurrence and resistance due to their ability to self-renew and adapt to treatments [60]. Brain cancers are characterized by high recurrence rates, particularly glioblastoma (up to 90% of patients suffering from grade IV GBM experience tumor regrowth) and WHO grade II/III glioma [61].

**Figure 2:**
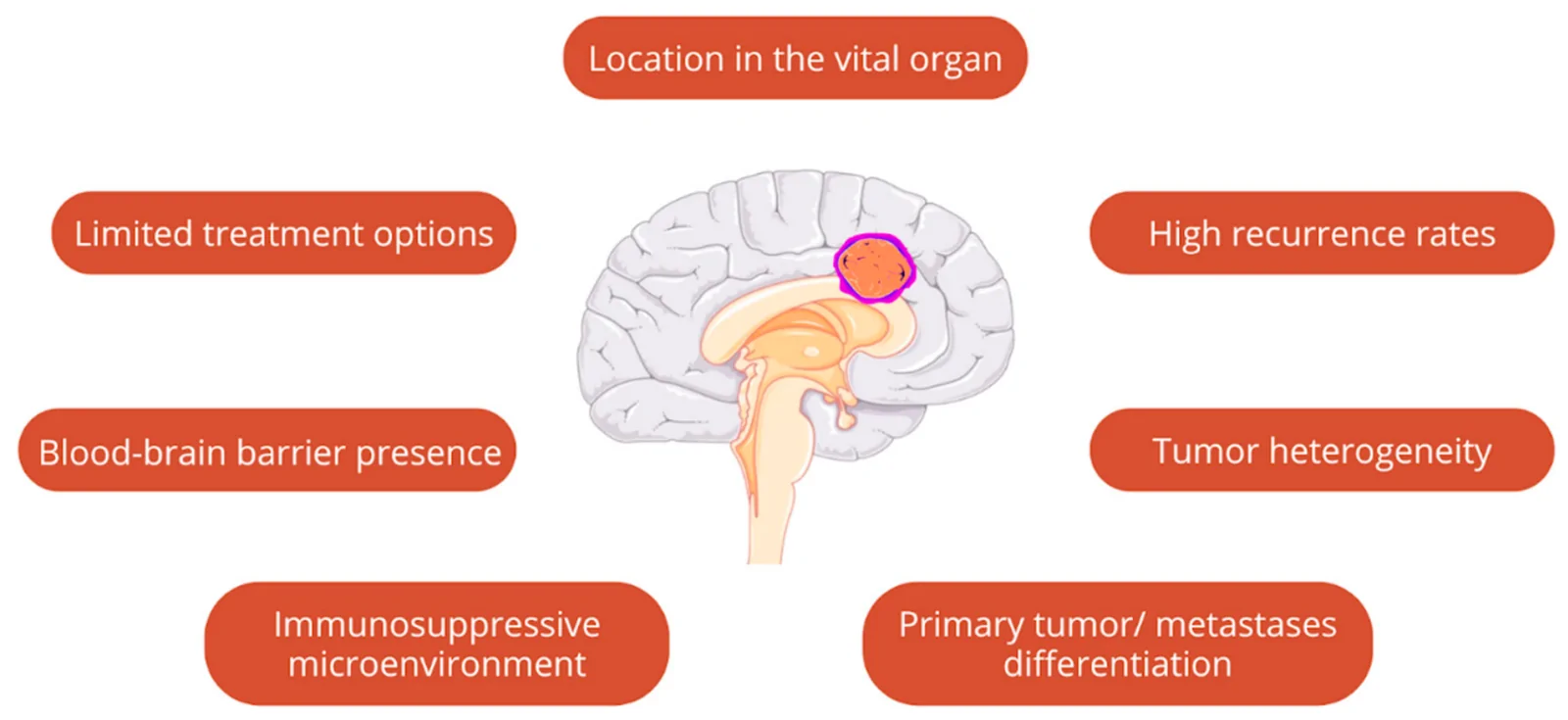
Major challenges in brain tumor treatment.

These two factors—BBB constraints and tumor heterogeneity—significantly limit the effectiveness of current brain cancer treatments, underscoring the need for novel strategies that can bypass these barriers and target diverse tumor cell populations effectively. What is also of note is that differentiating primary CNS tumors from secondary metastatic lesions can pose a challenge as well, which can have prognostic and therapeutic implications [62]. The above-described factors constraining the efficacy of brain tumor treatment are presented in Figure 2.

## 3. Plant Compounds with Therapeutic Potential Against Brain Cancers: Findings from Preclinical Studies

### 3.1. Curcumin (CUR)

Curcumin (CUR) is one of the bioactive curcuminoids, natural polyphenol compounds present in turmeric. This lipophilic polyphenol has gained popularity due to its various therapeutic properties, among which antioxidant, anti-inflammatory, antimicrobial, or antimutagenic can be listed [63]. Currently, much research confirms the anticancer role of CUR, including activity against brain tumors [64,65].

**Figure 3:**
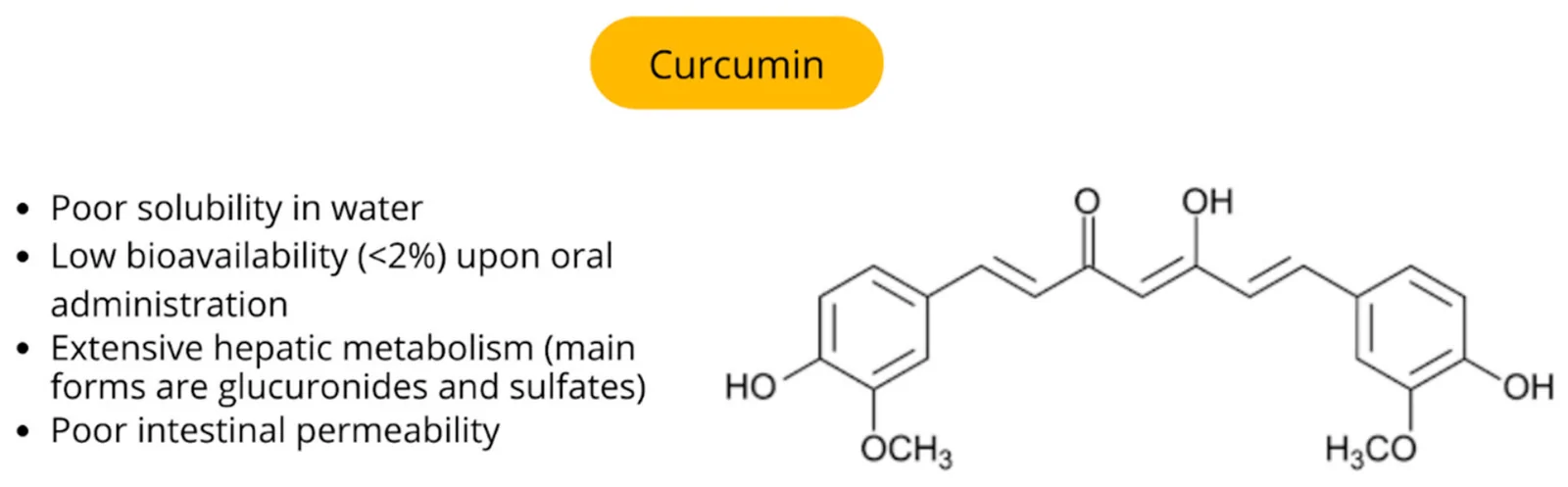
Structure and pharmacokinetic features of curcumin.

Structurally, curcumin belongs to the class of compounds known as diarylheptanoids. The molecule of curcumin is built of two substituted aromatic rings (o-methoxyphenol groups), connected by a seven-carbon linker containing an α,β-unsaturated β-diketone moiety (Figure 3). The latter tautomerizes between its diketone (keto) form and its more stable enol form; the keto form prevails in acidic to neutral pH (3–7), when hydroxyl groups are protonated, while at higher, basic pH values, curcumin exists as enol and exhibits better solubility in water. The diketone group not only serves as a scaffold enabling binding to various biomolecules but also chelates (forms stable complexes with) metal ions. The linker group contains unsaturated bonds, providing conformational flexibility important for the anticancer activity of curcumin (e.g., enabling reversible covalent interactions with proteins and binding to structurally unrelated targets, like kinases or transcription factors). The phenolic -OH groups and the methylene group adjacent to the diketone system donate hydrogen atoms to neutralize ROS, also contributing to the antioxidant role [66]. The main limitation for curcumin use in the clinic is poor bioavailability after administration (below 2%) [67].

#### 3.1.1. Mechanisms of Curcumin Anticancer Activity

CUR was found to decrease proliferation and promote the apoptosis of GBM cells via various pathways. Several studies reported an increase in pro-apoptotic protein Bax expression, together with reduced anti-apoptotic protein Bcl-2 levels after curcumin treatment. This consequently led to upregulated caspase-3 expression, suggesting the activation of the apoptotic pathway since caspase-3 is the major apoptosis executor [68,69,70]. Indeed, increased caspase-3 and beclin-1 protein levels were observed, indicative of apoptosis. Moreover, CUR was shown to downregulate in vitro the phosphorylation of Akt and mTOR, key components of pro-survival signaling pathways, which indicates a promotion of apoptosis in U251 and U87 glioblastoma cells. The phosphate and tensin homologue (PTEN) and p53 protein expression were upregulated in vitro and in vivo after CUR treatment, which led to inhibited growth and decreased volume of the tumor in vivo, since PTEN acts as a negative regulator of p-AKT/mTOR signaling pathway and p53 is a tumor-suppressing protein [68].

Curcumin’s anticancer effects are mediated by the downregulation or inhibition of a number of factors, for example, NEDD4, as observed in A1207 and SNB19 glioma cells [69]. The E3 ubiquitin-protein ligase NEDD4 is responsible for PTEN degradation by promoting ubiquitination and for PI3K/AKT signaling pathway activation, thereby maintaining proliferation. NEDD4 was found to boost invasion and cell migration, while its repression by CUR inhibited glioma cell proliferation, migration, and invasion [69]. The compound was also observed to downregulate the S-phase kinase-associated protein 2 (Skp2) pathway in U251 and SNB19 glioma cells, overexpression of which was found to promote cell migration and growth in various cancer cells, including GBM. Both mRNA and protein levels of Skp2 decreased after curcumin treatment, as did expression of pAkt. Simultaneously, levels of p57 tumor suppressor protein increased [70]. In another in vitro study on different cell lines (U138MG, U87, U373 and C6), Zanotto-Filho et al. also proved that curcumin displays pro-apoptotic effects on transformed cells [71]. It causes a decrease in the constant activation of PI3K/Akt and NFκB pathways, crucial for cells’ survival, impairs mitochondrial function and negatively regulates the antiapoptotic Bcl-xL protein.

Evidence of apoptosis induction by curcumin was also found in a study conducted in patient-derived glioblastoma stem cell (GSC) models. Treatment with CUR restricted the capability of GSCs to proliferate and reduced their sphere- and colony-forming capacity. At the molecular level, it increased the amount of phosphorylated form of ERK, as well as levels of p38 and the c-Jun transcription factor, indicating JNK and, consequently, MAPK activation. The inactivation of STAT3 through reducing phosphorylation at the Tyr705 position and an increase in phosphorylation at Ser727 was noted, making it not able to undergo nuclear translocation and thereby blocking its downstream effects. It is supported by the demonstrated decrease in anti-apoptotic proteins, such as survivin, a downstream target of STAT3, as well as protein inhibitors of apoptosis IAP1 and IAP2 in the studied cells. Some of these effects have been attributed to curcumin’s ability to induce ROS formation, since higher levels of ROS might have an anti-angiogenic effect through a decrease in the activation of signaling pathways like NF-κB [72].

Recent studies have indicated that another target of CUR is ENO1 gene-encoding enolase, the high expression of which promotes gluconeogenesis and the pentose phosphate pathway, as well as fructose and mannose metabolism. It was correlated with an increasing tumor grade of glioma. After curcumin treatment, ENO1 expression in GBM cells decreased, resulting in the inhibition of the glycolytic processes and, in consequence, less energy being produced. With an insufficient energy supply, the proliferation, invasion and migration of GBM cells were impaired and apoptosis was promoted [73]. Another mechanism through which CUR reduces the migratory capacity of GBM cells and lowers tumor invasion is inhibiting the expression of specific matrix metalloproteinases (MMPs), such as MMP-2, -14, -15, and -16 [74]. The discussed compound was found to block the phosphorylation of ERK1/2 in LN229 and U87 MG cells in vitro, with the subsequent inhibition of the exogenous norepinephrine (NE)-enhanced expression of MMP-2 and MMP-9. NE was provided to mimic the effect of psychological stress on the cells, which was recently studied to induce GBM cell proliferation. It was observed that curcumin inhibited NE-enhanced proliferation and could trigger apoptosis of these cells, which could occur via downregulation of anti-apoptotic proteins Bcl-2 and Bcl-xl. As a result, tumor cell proliferation and invasion decreased [75].

Curcumin’s activity also extends to epigenetic regulation, a theme highlighted in the Abstract of this review but, in the original literature search, underdeveloped in the body text. A 2025 study on the 1321N1 glioblastoma cell line found that curcumin nanoparticles (IC_50_ ≈ 1.7 µM) significantly downregulated DNMT3A (a de novo DNA methyltransferase), the Polycomb repressive complex 2 subunit SUZ12, the long non-coding RNA HOTAIR, and the methyl-CpG-binding protein MeCP2, alongside reduced invasion, growth, and migration of treated cells [76]. Because DNMT3A, SUZ12/PRC2, and HOTAIR jointly regulate chromatin compaction and gene silencing at tumor suppressor loci, this finding suggests that curcumin’s epigenetic effects may act as an upstream layer of regulation feeding into the transcriptional and signaling changes described throughout Section 3.1.1, rather than a separate mechanism; this connection has not, to our knowledge, been directly tested and is noted here as a hypothesis for future work.

In glioma in vitro cell lines, CUR was shown to induce apoptosis via cell-cycle arrest at G2/M phase [70,71] and G1 phase [77]. The compound was found to upregulate tumor-suppressing death-associated protein kinase 1 (DAPK1) mRNA and its expression that may induce G2/M arrest and apoptosis. Studies suggest that this effect may correlate with curcumin-induced STAT3 and NF-κB dephosphorylation, DNA-binding activity blockage and caspase-3 activation [78]. Another mechanism of such arrest induction could be the inhibition of the SHH/GLI1 (Sonic Hedgehog/glioma-associated oncogene homolog 1) signaling pathway, which is involved in cell-cycle progression. GLI1, as a transcription factor, regulates several target oncoproteins that influence cell cycle, survival, invasion, and self-renewal of cancer stem cells. CUR downregulated SHH, the Smoothened (SMO) transmembrane receptor, and GLI1 mRNA expression and inactivated Cyclin D1, Bcl-2, and Foxm1, which are GLI1-regulated oncoproteins and, at the same time, elements of the SHH/GLI1 pathway. The GLI1 expression was reduced in both the cytoplasm and the nucleus, suggesting dual activity of curcumin as a suppressor of GLI1 activation and its capability to prevent its nuclear translocation [70].

Moreover, curcumin was found to cause G2/M phase arrest not only in gliomas but also in human medulloblastoma cells. In the presence of curcumin, progression through the cell cycle is delayed or completely stopped in some cases. The mechanism of that was suggested to depend on the binding of curcumin to the cell division cycle protein 27 (Cdc27) with higher affinity to its phosphorylated form. This interaction seems to be linked with curcumin apoptosis promotion, as cells with high levels of Cdc27 phosphorylation were more susceptible to apoptosis induced by curcumin [79]. Beyond that, as shown in a 2013 study, CUR inhibited the anaphase promoting complex/cyclosome (APC/C) function by blocking the connection between APC/C crucial subunit Cdc27 and its activator—p55Cdc20. APC/C blockage results in G2/M arrest by the prevention of cyclin B1 degradation. Accumulated cyclin B1 prevents mitotic exit, locking cells in M phase [72]. Except for that, CUR was found to cause G1 phase arrest through downregulation of CCNE1, CDK2 and E2F1 genes, while upregulating suppressive PTEN expression. CCNE1 encodes for G1/S-specific cyclin-E1, and CDK2 encodes for Cdk2 kinases; a decrease in their levels can cause the mentioned cell-cycle arrest. A reduction in transcription factor E2F1 expression could disrupt the transcription of cyclin, CDKs, and a number of checkpoint regulators, further contributing to cell-cycle disruption or arrest [77].

It was found that curcumin causes cell death not only by inducing apoptosis but also via other cell death mechanisms. It was observed that treatment with curcumin-induced necrosis and apoptosis on the same level. The dead cell population and the early apoptotic one were close in quantity, which was tested by Thani et al. on U373 cell line [74]. An alternative mechanism for decreasing glioma cell viability by curcumin could be downregulating the expression of telomerase reverse transcriptase (hTERT), a catalytic subunit of human telomerase, thereby reducing its activity. Long-term curcumin treatment caused a shortening of the mean telomere length, supporting this hypothesis [74].

What could also be of use in anticancer therapies is the inhibitory role of curcumin on angiogenesis in different cancers [80]. However, the knowledge about the anti-angiogenic influence of curcumin in brain tumors is still limited and requires further research. The effect in gliomas was examined by Perry et al. in vivo on U-87 derived xenografts implanted into athymic mice. The excised tumor was pale pink, and the hemoglobin level was reduced, along with decreased expression of CD31 and CD105 mRNA, together confirming the antiangiogenic effect [81]. Both CD31 and CD105 are biomarkers for angiogenesis during vascularization, with the former being a molecule present on endothelial cells in general, and the latter being typical for newly formed tumor blood vessels. He et al. also observed downregulated CD31 expression after curcumin treatment, which was studied in vivo in the mouse glioma GL261 cell line [82]. The number of newly formed tumor microvessels was reduced as a result of treatment. Another mechanism by which formation of new vessels is inhibited after CUR administration is reducing the activity of matrix metalloproteinase 9 (MMP-9) and its pro-enzyme pro-MMP-9, since MMPs released by endothelial cells are known for their multifaceted role in neovascularization. Also, CUR reduced the proliferation of RBE4, a brain capillary endothelial cell line, which also indicates the anti-angiogenic effect of curcumin treatment in vitro. The inhibition of RBE4 cell migration and reduction in the width and length of formed endothelial tubular structures were noted. Both effects have been found to be dose-dependent [81].

#### 3.1.2. Analogs and Novel Formulations of Curcumin

The limitations related to poor aqueous solubility, rapid metabolic degradation, and limited bioavailability of curcumin in the central nervous system have triggered the development of structural analogs and advanced delivery formulations designed to enhance its stability, tissue distribution, and therapeutic efficacy. In particular, curcumin analogs aim to amplify its multitargeted biological activity while improving pharmacodynamic parameters, whereas novel formulations—including nanoparticles, liposomes, micelles, and conjugates—seek to overcome barriers related to systemic absorption and blood–brain barrier penetration.

Studies have shown that solid lipid curcumin particles (SLCPs) enhance the bioavailability and anticancer effects of curcumin in glioma, improving solubility and stability compared to natural curcumin [68]. Another work demonstrated that NRP-1-targeted exosomes loaded with SPION and curcumin effectively cross the blood–brain barrier, enabling simultaneous imaging and therapy of glioma in vitro and in vivo. The combined treatment showed strong antitumor effects and translational potential [83]. Furthermore, curcumin analogs such as EF24, which possess improved bioavailability and enhanced bioactivity, including anticancer, anti-inflammatory, and antibacterial effects, are being actively explored. EF24 inhibits tumor growth by inducing cell-cycle arrest and apoptosis, primarily through suppression of the NFκB signaling pathway and regulation of gene expression via microRNAs and the proteasomal pathway [84]. However, some studies have relied solely on in vitro data without in vivo validation. In vitro results do not always predict in vivo outcomes accurately, as they may fail to reflect crucial factors, such as immune responses and toxicity profiles [85,86]. Therefore, in vivo studies are essential before human trials can begin [85].

### 3.2. Resveratrol

**Figure 4:**
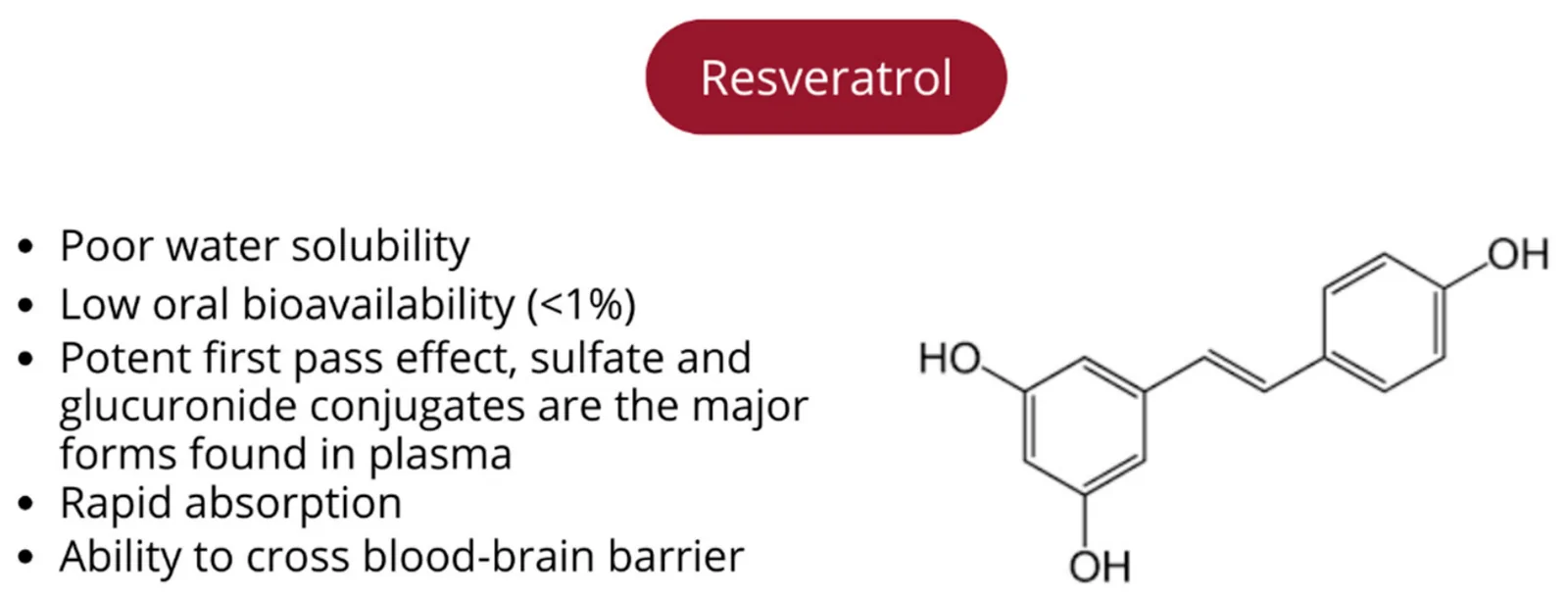
Structure and pharmacokinetic features of resveratrol.

One of the most widely studied plant-derived compounds in the context of its potential benefits for human health is resveratrol (RES). Chemically, it is a stilbene derivative (3,5,4′-trihydroxystilbene), belonging to the natural non-flavonoid polyphenol family. The core of the RES structure is two benzene rings linked by an ethylene bridge, with three hydroxyl groups attached to the rings at positions 3, 5, and 4′ (Figure 4). Due to the presence of the central double bond, two isomers are distinguished: trans—the most stable one, commonly found in plants, and cis, formed when trans-resveratrol is exposed to UV light or heat. Resveratrol can be found in many plant species and is particularly abundant in dark fruits, such as grapes and blueberries, but considerable amounts are also present in peanuts and cocoa [87,88]. It is produced by plants as a response to stress caused by pathogens, injury, or UV irradiation; the biosynthetic pathway leads to the synthesis of its trans form [22]. Due to its strong antioxidant properties, RES is an established component of numerous cosmetic products; its ability to modulate certain signaling pathways and regulate cell-cycle, proliferation and migration places RES among compounds of great potential to be exploited in anticancer therapies, including brain cancers.

#### 3.2.1. Molecular Mechanisms of RES Anticancer Activity: General Conclusions and Findings from Brain Cancers Cell Culture and Animal Studies

Resveratrol has been shown to interfere with cell-cycle regulation, autophagy, and apoptosis, which could be exploited in cancer therapy. The capability of RES to cause cell-cycle arrest was observed in various cancer types, with varying effects across cell culture types. For instance, in oral squamous cell carcinoma cell lines, RES-induced arrest at the G2/M transition phase in a concentration- and time-dependent manner, while in lung and liver cancer cells, an arrest at the G1 phase was observed through suppression of cyclins D1, D2 and E expression. G1/S-phase arrest was induced by RES in colon cancer and anaplastic thyroid carcinoma cells, also mediated by cyclin D1 depletion and modulated adenosine receptor activity [21].

The role of RES in triggering cell death is mainly based on p53 activation through the direct stimulation of sirtuin 1 (SIRT1) activity or domain-containing lysine methyltransferase 7/9 (SET7/9). SIRT1 deacetylates p53 at key lysine residues, thereby reducing its nuclear transcriptional activity. Accumulated in the cytoplasm, p53 can translocate to mitochondria, where it promotes apoptosis through transcription-independent mechanisms [89,90]. Additionally, RES is involved in cytokine-mediated apoptosis induction. As shown, treatment with RES is followed by pro-inflammatory cytokine release, an increase in NK cells’ population, and the downregulation of immunosuppressive cytokine expression, such as TGF-β. Induction of apoptosis and cell-cycle arrest is also mediated by STAT-3 suppression by RES, which leads to Bcl-2 and cyclin D inhibition [89]. The hypothesis that RES could induce apoptosis through direct interaction with death receptors remains yet unresolved [91]. In addition, a contribution of RES to mitotic catastrophe induction has been reported based on a study on glioma cells [92]. The effect of RES-mediated sensitization of cancer cells to other apoptosis inducers has also been reported [91]. Some reports indicating that RES can directly target mitochondrial respiration can also be found, stating that treatment with this compound increases the rate of OXPHOS and stimulates Ca2+ ion influx, contributing to apoptosis [93].

Resveratrol has a major impact on autophagy—a process of degradation and the recycling of cytoplasmic components, which, in cancer cells, largely contributes to their survival. However, depending on the cellular context, autophagy can also be lethal when overly activated. It has been found that RES leads to cell death in this mechanism through promoting autophagosomes’ formation, direct autophagy-inducing genes, like Beclin-1, Atg5 or Atg12 upregulation, or several signaling pathways. As a lipophilic compound, RES can cross the plasma membrane and modulate numerous signaling pathways inside the cell [94]. RES stimulates stress-activated kinases such as MAPK and Jnk, which further cooperate with Akt/mTOR suppression to enhance autophagic flux. Moreover, RES acts as an inhibitor of mTOR itself via AMP-activated protein kinase (AMPK) activation, triggering autophagic cell death. However, in lower concentrations, RES can induce only partial autophagy, which would act cytoprotectively [89,95]. Moreover, PI3K/Akt/mTOR is a major pathway controlling cell cycle, proliferation, and survival, integrating cellular signals that are transmitted through many signaling intermediates. It has been found that RES is an inhibitor of phosphorylated p110 and p85, subunits of PI3K. In consequence, PDK1 is inhibited and Akt does not undergo phosphorylation, impairing mTOR complex formation and thereby blocking cell proliferation [96]. Another molecular effect of RES is the downregulation of NF-κB expression—it prevents phosphorylation and degradation of κB inhibitor (IκB) by suppressing translocation of DNA chaperones like high mobility group box protein 1 [97].

Studies on glioma cells confirmed that RES in concentrations of 100 μM decreases both the expression and phosphorylation of Akt itself, blocking signal transduction through this pathway [96]. This stands in line with the results of a study authored by Clark et al., who demonstrated reduced Akt phosphorylation and upregulation of downstream p53 genes in patient-derived GBM stem-like cell (GSC) lines and U87 glioma cells. As a result, GBM and GSC growth and infiltration were significantly reduced, and similar effects were observed regarding mice xenografts of the studied models. These findings indicate that RES could be of use even in highly heterogeneous tumors [93]. An observation that Notch-1 is involved in RES-induced apoptosis, with the restoration of wild-type p53 expression as found in A172 and T98G GBM cell lines, is also worth mentioning [98]. A recent work shed light on another mechanism of RES activity against glioblastoma cells, which is the upregulation of PTEN, a well-known tumor suppressor. It acts as a phosphatase and negative regulator of PI3K/Akt signaling, suppressing the proliferation of cancer cells and enhancing their apoptosis [99].

Aberrant activation of the Wnt/β-catenin pathway is observed in multiple cancers, including brain malignancies. As a key network integrating and orchestrating crucial cellular processes including proliferation and stem cell phenotype maintenance, when overactivated, it significantly contributes to the renewal of cancer stem cells, supports cancer cells’ survival, and mediates their invasiveness through the EMT program [100]. Cilibrasi et al. performed a study investigating RES effect on seven patient-derived glioma cell lines. Although the responses to RES exposure highly varied among the studied cell lines, some general findings include downregulation of Wnt signaling and EMT-associated Snail and Twist transcription factors, as well as inhibited stem cell proliferation and reduced cell motility, as revealed by wound healing assays [101]. An older study reported that siRNA-mediated inhibition of Wnt and β-catenin induces apoptotic events in U-251 glioma cells and mouse models with a concomitant decrease in PI3K and phosphorylated Akt expression [102]. Certainly, more studies focusing on a thorough evaluation of the RES influence on various preclinical brain cancer models are needed to add a wider context to these results.

Interesting results were reported in 2013 by Sato et al., who have shown that RES is able to induce the differentiation of glioma stem cells (GSCs) into non-tumorigenic cells via the proteasome-dependent degradation of Nanog, mediated by RES-triggered p53 activation. In the study conducted on several patient-derived GSC cultures, RES inhibited their self-renewal and reduced their sphere-forming capacity, which—along with a decreased expression of stem cell markers that was also observed—is indicative of a loss of stemness features. The underlying mechanism was found to rely on the downregulation of Nanog protein expression and stability, a crucial one in the maintenance of GSCs [103]. Resveratrol’s qualities had also been researched regarding a crucial mitosis enzyme, telomerase. There is a specific subunit of this enzyme that is considered an “immortality” marker expressed in more than 85% of cancer cells. It has been proven in the U-87MG cells that RES administration decreases the cytoplasmic amount of this enzyme through the downregulation of its catalytic subunit mRNA expression, leading to cell division arrest. These findings further support the potential applicability of RES in anti-GBM therapies but need evaluation in vivo [104]. The therapeutic potential of RES against brain cancers was also assessed in animal models. A 2023 meta-analysis summarized 10 studies (nine in mice and one in rats), assessing the efficacy of RES intervention as a treatment of glioma and glioblastoma xenografts. Based on tumor volume measurements reported in the original studies before and after treatment, the therapeutic efficacy of RES has been proven both against xenografts formed by inoculation of human GBM cells, and rat glioma cells. In the majority of reports (eight out of 10), intraperitoneal administration of RES was chosen; however, the tumor volume reduction was similar in case of this route and single intravenous and oral administration reports. What is also valuable, the meta-analysis indicates, is that a significant tumor size decrease occurred only upon RES treatment lasting for at least 18 to 30 days [105]. Another research group also tested various routes of RES administration with mice bearing GSCs and GBM xenografts and found that intracranial delivery resulted in a 100-fold higher local RES concentration than after intravenous administration. However, RES placed in the water supply for animals—with solubility of 100 μg/ml—allowed them to attain a daily intake equivalent to 25 mg/kg per day, resulting in a significant reduction in average tumor volumes in the treated group. With a consideration of all RES-treated animals, regardless of route of administration, a general 40% inhibition in tumor growth was noted, underlying the rationale of further RES-based adjuvant therapies’ development [106].

As an example of a different approach, Song et al. studied the efficacy of RES as a complementary treatment after partial tumor removal from rats bearing orthotopic glioblastomas inoculated into their brains. The administration of RES via lumbar puncture every three days, starting from 24 h after tumor resection, resulted in tumor reduction and an increased lifespan in the study group in comparison to rats that had not received the compound. At the molecular level, RES was shown to increase apoptosis, suppress STAT3 expression and translocation into the nucleus, and mitigate tumor growth; however, no complete inhibition of the latter process has been observed [107].

#### 3.2.2. Bioavailability of Resveratrol in Context of Brain Cancers

The potential applicability of RES in supporting brain cancer treatment is possible due to its ability to cross the blood–brain barrier via tight junctions [108]. However, it has to be noted that RES exhibits poor bioavailability upon administration due to intensive metabolic processes in the intestine and liver, which contribute to its low plasma concentrations and thereby limited biological effect, particularly in the brain. Moreover, intestinal microbiota, responsible for RES metabolism in this part of the GI tract, is highly variable among individuals [22,109], further indicating that other routes of administration should be considered to enable RES to reach concentrations that would effectively inhibit tumor growth. The promising approach to administer RES in supporting brain tumor treatment is lumbar puncture, which is a standard administration route of therapeutics in this type of malignancies as a way to achieve high drug concentrations in a short time, with protection of healthy tissues and organs. Also, it allows for the application of lower drug doses in comparison to other administration routes. The successful lumbar puncture-mediated administration of RES for treatment of rat orthotopic glioblastoma has been reported, resulting in tumor size reduction and prolonged survival compared to the control group receiving saline. Effects induced by RES involved STAT3 signaling inhibition and the upregulation of LC3 and Beclin-1, resulting in autophagy and apoptosis induction. However, tumor growth was not suppressed completely, suggesting that combining RES with other therapeutic modalities would increase the success rate of anticancer treatment [110].

The improvement of RES bioavailability can also be achieved through the use of nanocarriers. Encapsulating RES in micelles, liposomes, polymeric nanoparticles or other formulations protects it from trans to cis isomerization and improves uptake, absorption, and thereby bioavailability when administered orally. One case study suggested that the delivery of RES through oral mucosa in the form of an adhesive film containing this compound can further increase its bioavailability, but obviously, this result cannot be regarded as standard [22]. A few studies assessing nanosystems in targeted RES delivery to brain tissue have been set in the context of brain cancers. For instance, transferrin-coated PGA-PLA nanocarriers containing RES, developed by Jhaveri et al. on the basis of transferrin receptors’ presence in the blood–brain barrier, were characterized by increased intracellular uptake and a higher rate of apoptosis induction in rat C6, human U-87 MG GBM cells, and a xenograft U-87 MG mouse model compared to free RES [111]. Recently, in a combined in vitro/in vivo study, it has also been proven that RES is an efficient tumor growth suppression agent. In the 2021 study, it was shown that the designed nanoparticles targeting a typical GBM marker deliver RES to the tumor sites. The issue of low bioavailability at the tumor site of RES was overcome by encapsulating it, delaying its release and therefore its action. A short peptide, Pep-1, has shown an affinity for GBM xenografts, making it a perfect component of the nanoparticle. Low toxicity to normal brain cells was also shown in the study. The development of nanoparticles (drug carriers) improves the drug’s retention in the tissue and final outcome. The number of proliferating GBM cells decreased as the rate of internalization of the nanoparticles grew, which depended on the structure of the carrier. The effects were also longer-lasting compared to those of the control group [112].

Beyond delivery-focused reformulation, structural analogues of resveratrol have also been evaluated directly in glioma models. Pterostilbene, the dimethyl ether analog of resveratrol found in blueberries, has markedly better oral bioavailability than resveratrol itself and has already undergone a human safety trial for other indications. In T98G, LN18, LN229, U87, and rat C6 glioma cells, pterostilbene inhibited proliferation, induced intrinsic mitochondria-mediated apoptosis, caused S-phase arrest, and inhibited migration and invasion, with effects attenuated by a pan-caspase inhibitor, confirming a caspase-dependent mechanism [113]. A related methoxylated analogue, 3,5,4′-trimethoxystilbene (TMS), was directly compared with resveratrol and pterostilbene in C6 and T98G cells: resveratrol and pterostilbene promoted S-phase accumulation, whereas TMS induced G2/M arrest and was reported to be a more potent apoptosis inducer, suggesting that even closely related methoxylated stilbenes can differ in their cell-cycle mechanism, not only in potency. Because pterostilbene and TMS retain resveratrol’s core stilbene scaffold while substituting hydroxyl for methoxy groups, their improved bioavailability profile makes them attractive candidates for the brain-delivery strategies discussed above, and we see the systematic side-by-side testing of resveratrol, pterostilbene, and TMS under matched delivery conditions as a logical next step [30].

### 3.3. Berberine

Berberine (BBR) is an isoquinoline alkaloid present in various plants, firstly isolated from Xanthoxylon clava, known as pepperwood [20]. Initially used in traditional Chinese medicine, the compound turned out to have numerous beneficial applications, including antimicrobial, anti-diabetic, and lipid-lowering, among others. Recently, the number of studies suggesting berberine as a potential anticancer agent has grown, including research focusing on the treatment of brain tumors [20,100,101].

**Figure 5:**
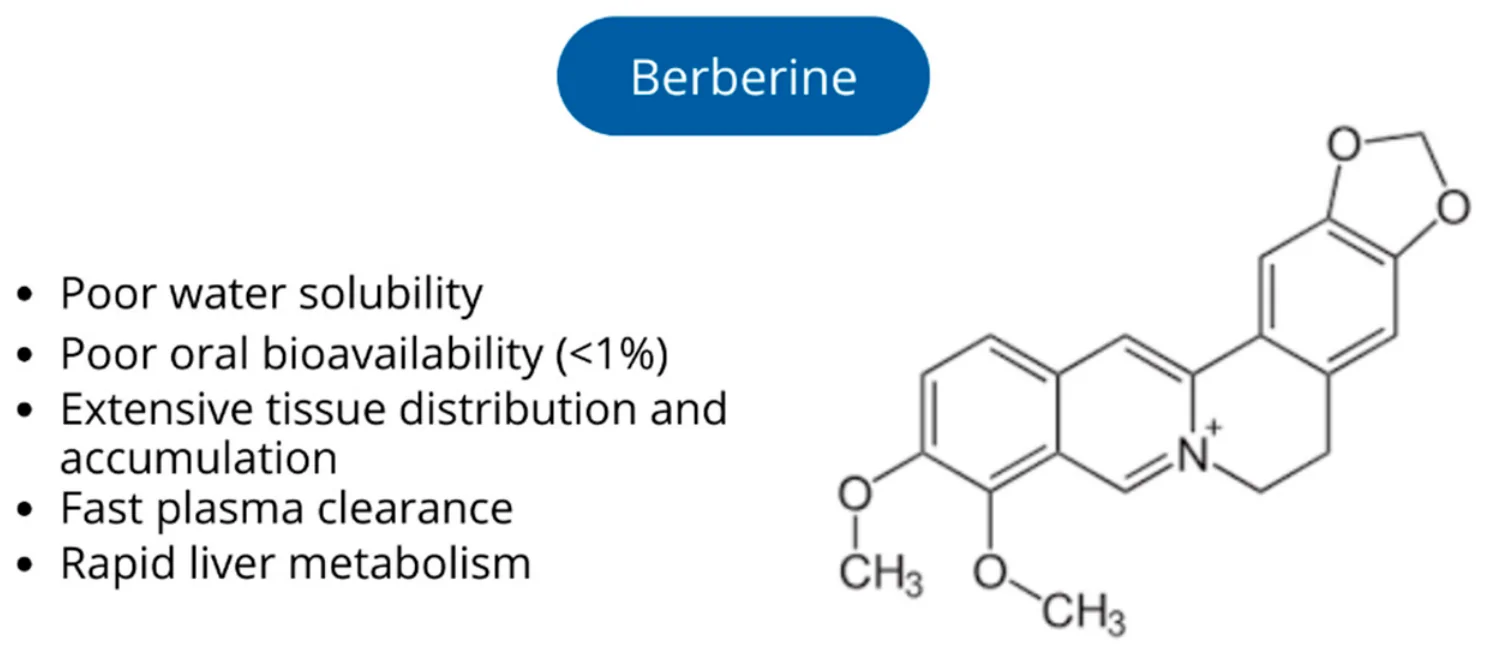
Structure and pharmacokinetics of berberine.

The core of the berberine structure is formed by a tetracyclic structure with a quaternary nitrogen atom (Figure 5). This positively charged nitrogen within the ring system is crucial for many of berberine’s biological actions, including antibacterial properties. Ring A is additionally endowed in a dioxolane ring, essential for lipid-lowering activity, while methoxy groups attached to ring D (position C-9 and C-10) are associated with antidiabetic properties of berberine [101,102].

Unlike curcumin and resveratrol (Figure 3 and Figure 4), berberine’s blood–brain barrier permeability is not self-evident from its structure and deserves separate comment. Berberine carries a permanently charged, quaternary nitrogen atom (Figure 5), which markedly limits passive transcellular diffusion across lipid membranes, including the BBB. In addition, berberine is a recognized substrate of P-glycoprotein (P-gp), an efflux transporter highly expressed at the BBB, which actively pumps the compound back into the vasculature and further restricts its net brain accumulation. Together with its already poor oral bioavailability (Figure 5), these two properties mean that berberine’s intracranial exposure after systemic administration is expected to be low, and this should be viewed as a significant, compound-specific translational obstacle on top of the general pharmacokinetic limitations discussed for all three compounds in Section 5.

A quantitative comparison analogous to those given for curcumin and resveratrol reinforces this point: oral berberine has a reported absolute bioavailability below 1% (Figure 5), and published human pharmacokinetic studies (conducted for its metabolic indications, not oncology) report peak plasma concentrations in the low nanomolar-to-sub-micromolar range after standard oral doses. This is orders of magnitude below the 10–50 µM range used in most of the in vitro glioma studies summarized in Section 3.3. As with curcumin and resveratrol, this gap does not invalidate the mechanistic findings, but it does mean that systemic oral dosing of unmodified berberine is very unlikely to achieve anti-glioma concentrations in the brain, and delivery innovation is a prerequisite—not an optional enhancement—for clinical translation. Structural analogues of berberine have also been developed, though largely outside the glioma literature to date. Systematic structure-activity work has focused on substitutions at the C-9 and C-13 positions of the berberine scaffold: 9-O-alkylated and 9-/13-disubstituted derivatives with extended lipophilic side chains have shown markedly improved antiproliferative potency compared with unmodified berberine—in some series, 48- to 125-fold lower IC50 values—in hepatocellular, colon, prostate, and breast cancer cell lines [114]. To our knowledge, these potency-optimized analogues have not yet been tested in glioma models specifically, which we see as a notable gap, given how much more potent they are than the parent compound in other tumor types. On the delivery side rather than the structural side, a 2026 study described folate receptor-targeted bovine serum albumin nanoparticles for berberine, designed to exploit folate receptor overexpression on glioblastoma cells; these nanoparticles enhanced intracellular uptake and suppressed 3D glioblastoma spheroid formation more effectively than free berberine [115], representing a targeted delivery complement to the lipophilicity-based structural modifications described above.

Berberine changed the expression and activity of apoptosis-related factors towards cell death induction. In several studies, a decrease in antiapoptotic Bcl-2 protein level and an increase in pro-apoptotic Bax were observed in BBR-treated GBM cells, as examined in human U251, U87, P3, T98G, and rat-derived C6 cell lines. This altered Bax/Bcl-2 ratio leads to mitochondrial membrane permeabilization and increased cytochrome C release, which directly promotes apoptotic cascade activation, in U251, U87 and C6 cells. Increased active caspase-9 levels in T98G and C6 cells, PARP activity in the former, and upregulated caspase-3 activity in all mentioned cell lines have been noticed. These higher levels of further effectors of the apoptotic pathway led to decreased cell viability. BBR was proposed to facilitate activation of apoptosis signaling also via upregulating the death receptor Fas (CD95) expression on the surface of C6 cells [116,117,118,119,120].

Another mechanism of cell death promotion by the discussed alkaloid is the inhibition of the AMPK/mTOR/ULK1 pathway, as shown in U251 and U87 GBM cells. BBR increases phosphorylation of AMPK and, as a result, downstream suppression of mTORC1 occurs, evidenced by a decrease in Ser-2448 phosphorylation of mTOR. Since mTORC1 is known to maintain an inactive state of Unc51-like kinase 1 (ULK1), its inhibition, together with the direct phosphorylation of the kinase by AMPK, promotes its activity. Consequently, ULK1 activates autophagy-inducing Beclin-1 complexes. This autophagy enhancement was suggested to induce toxic accumulation of autophagosomes, reducing proliferation and probably leading to apoptosis. Increased p-AMPK levels and lowered p-mTOR were found in vivo as well, confirming the cell-based findings [121].

BBR induces cell death through ER stress and the mitochondrial-dependent apoptotic pathway associated with ROS. Twelve h long treatment with this compound resulted in increased phosphorylation of PERK (PKR-like ER kinase) and eIF2α, but 18–24 h long treatment reduced this effect in T98G cells [120]. The expression of GRP78, a major ER chaperone responsible for protein quality control, and transcription factor CHOP were found to be upregulated in C6 and T98G cells [119,122]. These results suggest berberine-triggered promotion of ER stress, which leads to increased Ca2+ levels and consequently boosts ROS production [122]. In U87 cells, berberine enhanced not only the generation of ROS but also lipid peroxidation products, referred to as TBARS (thiobarbituric acid reactive substances) and protein carbonylation rates, consequently causing a release of cytochrome C and inducing apoptosis. Interestingly, catalase and superoxide dismutase activities were upregulated, and glutathione peroxidase activity was downregulated in berberine-treated U87 cells. However, the levels of antioxidant enzymes were insufficient to reduce oxidative stress [123].

Cell-cycle arrest could be another mechanism of the BBR-induced antiproliferative effect [119,120,123]. The accumulation of cells in G1-phase and decrease in cell number in G2/M phase was observed after berberine treatment on T98G and U87 cell lines [120,123]. In some cases, a decrease in the cell number during the S-phase was also reported. The observed upregulation of p27 protein expression and downregulation of CDK2, CDK4, and cyclins D and E were suggested as the mechanism of such arrest [120]. Another study on C6 cells also revealed an arrest of G2/M phase with a significant decrease in cells in S-phase. Observed reduced levels of cyclin B, Cdk1 and Cdc25C phosphatase, along with the upregulated expression of Wee1, a Cdk1 inhibitory kinase, were suggested as the plausible underlying cellular mechanism. With an increasing BBR concentration, the number of sub-G1 cells increased, suggesting consequent apoptosis induction [119]. Another study revealed different effects of berberine on cell cycle and proliferation, depending on the TP53 variant, a gene often mutated in cancer cells, changing tumor-suppressive wild-type p53 (wtp53) into pro-proliferative mutant p53 protein (mutp53). In non-mutated U87 cells, upon treatment with BBR, the number of cells in the G1 phase increased, which was accompanied by enhancement of wtp53 phosphorylation. Also, the authors observed inhibition of glioma cell proliferation mediated by BBR through the p53/p21/cyclin D1 pathway. On the other hand, for cells with mutated p53, no such effects have been noticed; however, BBR promoted the degradation of mutp53 in U251 cells, along with an observed increase in the cell number during the G2 phase. These results suggest that berberine induces wtp53 phosphorylation and lowers the mutp53 level, enhancing cell-cycle arrest and decreasing proliferation [124].

BBR was suggested to induce cell death not only via apoptosis but also oncosis [125] and to direct cells towards senescence state [126]. Oncosis is a form of cell injury leading to death, including whole-cell and organelle swelling, accompanied by increased permeability and the blebbing of the plasma membrane, correlated with decreased ATP levels [127]. Sun et al. have noticed that exposure to berberine results in changes characteristic of oncosis in T98G and LN18 cells: cell swelling, increased vacuolation originating from mitochondria, loss of organelles, and a membrane surface rich in blebs. Neither caspase-3 nor PARP-1 cleavage accompanied these morphological changes, which, along with reduced ATP production, excludes the possibility that apoptosis occurred and indicates oncotic cell death. Based on observed mitochondrial depolarization and decreased aerobic respiration rate, mitochondrial dysfunction induced by decreased ERK activity was proposed as a mechanism of oncosis promotion—berberine treatment reduced ERK1/2 phosphorylation, as observed in T98G, LN18 and C6 cells as well as in vivo in nude mice and rats [125]. This reduced the activation of ERK1/2, which is known as a mediator of proliferation in cancer cells, and is also one of the suggested mechanisms of inhibiting glioma cell proliferation [126]. Other signs of mitochondrial dysfunction leading to oncosis were decreased levels of GSH and NADPH after 48 h long berberine treatment, since GSH (reduced glutathione) is known for its function as the intracellular ROS scavenger and NADPH is crucial for restoring this enzyme [125].

Apart from oncosis, senescence induction was promoted by berberine in GBM, based on the in vitro studies in U87, U251, and U118 cells. Based on studies involving an EGFR inhibitor, the authors proposed that entering the senescence state was mediated by the downregulation of the EGFR-MEK-ERK signaling pathway, known to support cellular proliferation. A berberine-induced decrease in EGFR expression suppresses downstream signaling, subsequently inhibiting signal transduction through the MEK-ERK pathway, which disrupts cell proliferation and growth [126]. BBR was also observed to inhibit angiogenesis in U87 and U251 human glioblastoma cells via the VEGFR2/ERK pathway. The VEGFR2 phosphorylation, crucial for activating downstream signaling pathways that lead to formation of new vessels, was reduced, as well as phosphorylation of ERK and p38. The results were supported by lower vascular density in vivo and inhibited cell migration in vitro, along with lowered expression of an angiogenic biomarker, CD31. Together, all of this indicates that berberine inhibits vascularization of GBM in vivo. Additionally, the survival rate of mice in the orthotopic model was improved—BBR treatment reduced tumor volume and decreased hemoglobin level in ectopic models [128].

Notably, BBR is able to suppress glioma cell migration ability via inhibiting the epithelial–mesenchymal transition (EMT). Cells exposed to this compound exhibited the upregulation of epithelial markers β- and α-catenin, while the expression of mesenchymal markers such as vimentin and α-SMA was downregulated in U251 and U87 GBM cells, suggesting a suppression of the EMT process [129]. Another mechanism of inhibiting migration and invasion by the discussed agent was proposed to be energy generation disruption. BBR decreased the ATP and L-lactate levels and reduced the oxidative phosphorylation rate. More fragmented mitochondria were found in berberine-treated U251 and U87 GBM cells, indicating that it also induces alterations in mitochondria morphology, influencing their activity. Also, F-actin filaments of GBM cells exposed to berberine were less organized than in untreated ones, suggesting impaired cell migration [121].

Berberine was found to lower the weight of glioma tumors in vivo. Experiments conducted on nude mice with subcutaneous glioma xenografts revealed a significant decrease in tumor growth, and the effect was time-dependent. Simultaneously, no body weight loss was observed, which is an important finding since it suggests only tumor shrinking [121]. In another in vivo study, where glioblastoma xenografts were inoculated into the right striatum of mouse brains, a reduction in tumor growth after treatment with berberine was noted as well. Similarly to previously mentioned studies, BBR did not cause any decrease in body weight, indicating a tumor-specific effect [128].

**Figure 6:**
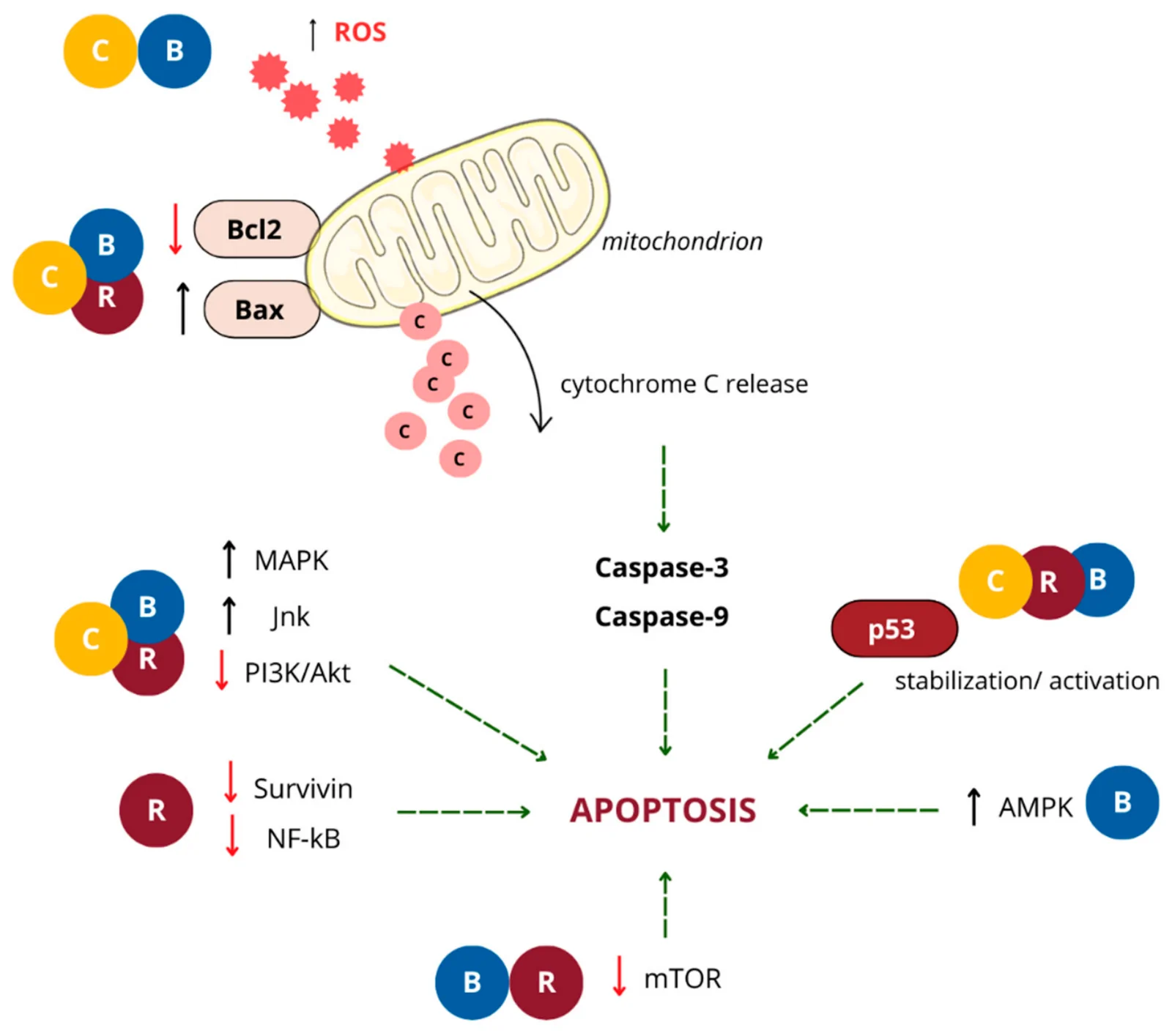
Resveratrol (R), curcumin (C) and berberine (B) induce or enhance apoptosis through numerous mechanisms. For example, all of these compounds upregulate the pro-apoptotic protein Bax and have an opposite effect on anti-apoptotic Bcl-2. This initiates intrinsic apoptosis through release of cytochrome c from mitochondria, in response to which initiator and executioner caspases are activated. Red downward arrows represent reduction, while black upward arrows—increase in expression of certain molecules. Green dashed arrows represent activation.

Figure 6 depicts mechanisms in which the three discussed compounds—resveratrol, curcumin and berberine—induce apoptosis in brain-derived cancer cells, as discussed in Section 3.

Evidence-level caveat: this figure intentionally aggregates findings across in vitro, in vivo, and (where available) ex vivo patient-derived models to show the overall convergence of mechanism across the three compounds, but it does not visually distinguish which specific arrow was demonstrated in which type of model. Readers should treat this figure as a synthesis of the mechanism, not as an indication that every pathway shown has been confirmed at every level of evidence for every compound.

## 4. Combinations with Conventional Treatments

A methodological note on terminology: few of the studies discussed in this section formally established synergy using a combination index, isobologram, or Bliss/Loewe independence analysis. Where the original authors reported such an analysis, we retain the term “synergy/synergistic”; elsewhere, we use the more cautious descriptors “enhanced” or “greater combined effect” to avoid overstating the strength of the pharmacological interaction.

### 4.1. Combining Plant Compounds with Chemotherapy: Outcomes and Effects

Studies highlight the potential of natural substances not only as direct anti-cancer agents but even more as part of combination therapies to enhance the effectiveness of existing treatments and reduce side effects. In the case of most brain cancer types, adjuvant chemotherapy after maximal safe resection is recommended with temozolomide (TMZ), a dacarbazine derivative, as a first-choice agent. TMZ is a prodrug, converted spontaneously to its active form at the physiological pH of blood. As an alkylating agent, TMZ attaches electrophilic alkyl groups to DNA, primarily at the N7 and O6 positions of guanine residues and the N3 position in adenine, disrupting the DNA structure and causing damage to it. A key feature of TMZ, enabling its use in brain cancer treatment, is its small size, together with high lipophilicity, due to which its molecules can cross the blood–brain barrier. Moreover, TMZ is characterized by fast absorption and good tissue distribution [130]. However, the upregulation and modulation of DNA repair mechanisms in cancer cells, along with activation of methyltransferases and increased drug efflux, often mediate resistance development, limiting therapy efficacy [131]. Beyond TMZ, several other cytotoxic agents are used in brain cancer treatment strategies, including nitrosoureas that induce DNA double-strand breaks (lomustine, carmustine); a PCV regimen based on a combination of procarbazine, lomustine, and vincristine; and cyclophosphamide and methotrexate as a standard for primary CNS lymphoma [132].

#### 4.1.1. Curcumin

Owing to its antitumor effects presented in Section 3.1, the combination of curcumin and TMZ against various brain cancer cell lines was evaluated and found efficient in several in vitro and animal studies. Zanotto-Filho et al., in their 2015 research article, indicated that mechanisms underlying the enhanced cytotoxicity of the combination regimen, assessed on three GBM cell lines and mouse C6 cell brain implants, include the inhibition of STAT3, NF-κB, and PI3K/AKT, with the activation of the G2/M checkpoint common for both curcumin and TMZ, via phosphorylating key proteins such as Cdc2 or Cdc25c. Notably, co-treatment was harmless to astrocytes. Moreover, the authors found that protective autophagy was induced by both compounds through ERK1/2 signaling, leading to DNA damage response and repair, and indicated that there is a rationale for including an ERK inhibitor to increase TMZ/curcumin efficacy [133]. Another study showed that curcumin can also sensitize GBM cells to TMZ through miR-146a upregulation and NF-κB inhibition, complementing the checkpoint activation leading to apoptosis [134]. An additional reported mechanism mediating the sensitization of U87MG glioblastoma cells to TMZ by curcumin has been ROS generation and AKT/mTOR signaling disruption, more potent upon exposing cells to combination therapy than to curcumin or TMZ alone [135].

TMZ was not the only chemotherapeutic that curcumin was combined with in preclinical studies. In a 2007 study, pretreatment with curcumin increased the susceptibility of T98G and U87MG cells to damage induced by cisplatin, etoposide, camptothecin, and doxorubicin, significantly increasing DNA fragmentation [136]. An enhanced cytotoxic effect has been reported for co-treatment with paclitaxel and curcumin; moreover, this combination reduced the migratory capacity, along with the expression of survival, proliferation, and angiogenic factors of GBM cells [137]. Also, curcumin and its nutraceutical formulation, known under the brand name of turmeric force (TF), were able to enhance the toxicity of etoposide and TMZ towards U87MG glioblastoma and D283 medulloblastoma cell lines. D283 cells were much more sensitive to the treatment than GBM cells, but in both cases, TF addition increased the cytotoxic effect of etoposide and TMZ more strongly than the conventional formulation of curcumin did. The pro-apoptotic effect, evidenced by an increase in the Bax/Bcl-2 mRNA ratio, was most pronounced when TMZ, etoposide, and TF were combined together [138].

#### 4.1.2. Resveratrol

Cell lines and animal studies strongly suggest that RES can be a potent ally during brain cancer chemotherapy. Particularly widely, an enhanced effect of combining RES with TMZ compared to TMZ alone is reported, as validated in cell lines and animal models; the broad range of biological effects of RES, presented in detail in Section 3.2, allows it to enhance anticancer activity of the standard chemotherapeutics. Yuan et al. found that such augmentation of TMZ anticancer effect relies on RES-triggered increased ROS generation, leading to AMPK activation and the following Bcl-2 downregulation, contributing to increased pro-apoptotic effects of TMZ in human GBM cell line SHG44. Moreover, the combination of RES and TMZ caused cell-cycle arrest in G2/M phase, caused a decrease in Ki67 proliferation marker expression and upregulated the astrocyte differentiation marker GFAP (glial fibrillary acid protein) [139]. A more recent study evaluated the effect of RES and TMZ in various concentrations on rat and human GBM cell lines. Although the low-dose RES-TMZ treatment suppressed the growth of rat RG-2 cells, only higher concentrations of both compounds (75 and 750 μM, respectively) were efficient with human LN-18 and LN-428 cells. However, suppression and apoptosis rates differed significantly between cell lines, with LN-18 responding better to the applied treatment than LN-428. The benefit of including RES in the treatment was MGMT downregulation and STAT3/Bcl-2/surviving pathway inhibition [140].

Apart from an enhanced effect of RES in TMZ-based therapy, it has been shown that this compound can also increase sensitivity of brain cancer cells to chemodrugs. One of the reported mechanisms of such sensitization, validated in T98G and U138 cell lines, is downregulation of MGMT, whose overexpression is the most pronounced mediator of TMZ resistance [141,142]. RES has also been found to be effective with glioblastoma-initiating cells (GICs)—a population exhibiting stem-like properties, largely responsible for tumor progression and chemotherapy resistance development. On patient-derived GICs, used to establish in vitro cultures and injected into mice, Li et al. have demonstrated that RES enhances TMZ-induced apoptosis by causing DNA double-strand breaks and promoted the differentiation of the cells via STAT3 inactivation [143]. However, despite promising results both in vitro and in animals, there are no current human trials registered at ClinicalTrials.gov assessing RES efficacy in brain and central nervous system tumors.

Some novel therapeutic approaches developed against GBM include RES with preclinical success. In one study, treating U-87 MG human glioblastoma cells with 10 and 15 μM RES, in combination with Hsp27-targeting siRNA or without it, for a duration of 48 h led to downregulation of heat shock factor-1 (HSF-1) and Hsp27, and an increase in caspase-3, indicative of apoptosis. This effect was suggested to depend on blocking phosphorylation and trimerization of HSF-1 and thereby lowered Hsp27 gene expression, which can be related to RES capability of blocking MAPK, Akt and mTOR. Although the most pronounced effect occurred when 15 μM of RES was combined with 25 nM of siRNA, some statistically significant effect of RES alone was also noticeable [144]. In another study, RES was incorporated into liposomes along with epirubicin to improve their transport through the blood–brain barrier, modeled in vitro and in vivo in C6 glioma-bearing rats. Observations included robust drug delivery into glioma cells, uptake, and apoptosis induction; animals in the study group also presented a significantly longer median survival time. These results suggest both the use of targeted liposomes and epirubicin as potentially beneficial in glioma treatment [145].

#### 4.1.3. Berberine

Unlike curcumin and resveratrol, BBR has not been extensively evaluated in the context of improving brain cancer chemotherapy efficacy. However, it would be reasonable to assess its potential combined effect with certain chemotherapeutic agents due to reported molecular activities of berberine, such as suppression of EGFR-RAF-MEK-ERK signaling, which can provide additional therapeutic effects and might increase overall efficacy since neither TMZ nor etoposide and nitrosureas directly act on this pathway. Yet, berberine is considered rather as a potential neuroprotective compound that may support chemotherapy and mitigate its toxicity towards healthy neural cells [114,146].

### 4.2. Plant-Derived Compounds and Radiotherapy

The search of scientific literature reveals that RES can have a positive effect on radiotherapy efficacy. Wang et al. performed a dual in vitro and in vivo study to assess the potential of RES as a radiation sensitizer. The in vitro part was conducted on a glioma stem and progenitor cell line SU-2, treated with RES and/or exposed to ionizing radiation; combined treatment resulted in lower colony and sphere formation capacity of GSCs, lower stemness marker expression, and apoptosis induction. Moreover, radiation-induced DNA damage repair was impaired upon RES exposure. In vivo validation (nude mice GSC xenografts) confirmed the autophagy- and apoptosis-inducing effects of RES, as well as tumor volume reduction [147]. A 2022 study in rats with an intracranial GBM model from the C6 cell line confirmed the potential of RES as an RT sensitizer, as indicated by a reduction in mortality and decreased tumor volumes mediated by RES-induced GBM cell-cycle arrest, cancer cell DNA damage, and apoptosis. Notably, RES treatment alleviated damage to healthy brain tissue in terms of cancer cells’ infiltration and radiation and improved the cytokine profile and immunomodulatory effect. The authors also reported a reduction in DNA damage in splenocytes of rats treated with RES and radiotherapy in combination, indicating pleiotropic beneficial effects of this compound [148]. Moreover, the ability of RES to inhibit STAT3 signaling is also beneficial in the context of RT. This transcription factor is upregulated in response to radiation, promoting cell survival and proliferation, which may eventually lead to the development of radioresistance. It has been previously shown, e.g., in glioma animal models, that inhibiting STAT3 can enhance the efficacy of RT and overall treatment [149]. A number of studies, including those mentioned in previous sections concerning RES anticancer activity, demonstrated the suppression of STAT3 by this compound in vitro and in vivo, with a mechanism based on inhibiting Tyr705 phosphorylation and, in turn, phosphorylating Ser727 of the STAT3 molecule, related to a loss of its activity [150,151]. In GBM-specific models, resveratrol was shown to suppress self-renewal and the tumor-initiating capacity of patient-derived glioblastoma stem cells (GSCs). Experimental data revealed the activation of TP53 and upregulation of p21, accompanied by the downregulation of stemness markers such as SOX2 and nestin. Furthermore, resveratrol promoted the differentiation of GSCs and significantly increased radiosensitivity in radioresistant glioblastoma cell lines, suggesting its potential as a radiosensitizing agent capable of overcoming therapy resistance [103,151].

Curcumin also presents potential in increasing the efficacy of RT. An enhanced antiproliferative effect was reported by Zoi et al., who exposed GBM cells of U87 and T98 lines to CUR prior to irradiation with 2 and 4 Gy. The combined treatment increased tumor cell death, mainly through cell-cycle arrest in G2/M phase, which was greatly enhanced by curcumin [152]. These cell lines were also used in a study using high and low linear energy transfer radiation therapy in order to induce radiosensitization. Application of CUR led to cell-cycle arrest, greater ROS production, and an increased apoptosis rate; combined with neutron irradiation, curcumin contributed to inhibition of cell invasion [153]. The ability of curcumin to suppress Hedgehog signaling was found to be related to increased efficiency of γ-irradiation (6 Gy), as studied on glioma cell lines [154]. These radiosensitizing effects of CUR in vitro have been confirmed by several animal model studies. A 2020 study showed enhancement of RT suppressive effect on growth of both in situ brain tumors and orthotopic glioma cell-based xenografts in rats [155]. In nude mice with intracranial LN-229 glioma models, subjected to 60 mg/kg CUR pretreatment and 18 Gy RT, such an intervention led to significantly higher tumor volume reduction in comparison to the control and RT-only groups. However, this effect was not observed in the case of U251 cell-derived tumors. An immunofluorescent examination of tumor tissues formed from both cell types showed the downregulation of the mesenchymal marker vimentin with an increase in E-cadherin and epithelial cell marker expression, suggesting a role of curcumin in attenuating the invasiveness of cancer cells [154]. Despite these encouraging results, it must be noted that doses of curcumin used in preclinical trials do not really match the concentrations achievable in the clinical setting. Importantly, the study by Sminia et al. revealed that clinically achievable concentrations of CUR did not affect the U251 human glioma cell survival exposed to both single-dose and fractionated radiation, showing that, unless in novel formulations, curcumin would not improve radiotherapy effects for GBM patients [156].

### 4.3. Potential of Curcumin and Resveratrol to Bypass or Reverse Drug Resistance

Drug resistance remains a major obstacle in effective cancer therapy and is a principal cause of treatment failure and tumor recurrence, particularly in highly aggressive brain tumors such as glioblastoma. Experimental and translational studies indicate that resistance mechanisms involve cancer stem cell (CSC) maintenance, dysregulated cell-cycle control, enhanced DNA repair, and altered apoptotic responses, underscoring the need for agents capable of targeting these processes [157,158].

Resveratrol has been extensively investigated for its ability to overcome drug resistance by targeting cancer stem cells and non-coding RNAs. In vitro and in vivo studies, summarized in a review by Zhou et al. [132], demonstrated that resveratrol modulates the expression of multiple miRNAs (including miR-34a and miR-21) and lncRNAs involved in stemness, survival, and chemoresistance. These regulatory effects led to reduced CSC viability, impaired self-renewal, and increased sensitivity of resistant cancer cells to chemotherapeutic agents. Although RES exhibited significant anti-CSC activity, the authors emphasized that its limited bioavailability remains a major barrier to clinical translation [158]. Several studies report that RES is able to restore the sensitivity of gastric and breast cancer cells resistant to doxorubicin, and glioma/glioblastoma cell lines resistant to temozolomide. In a recent study, it was shown that RES increased TMZ toxicity against resistant U251 cells through MAPK inhibition and P-glycoprotein downregulation [159]. The mechanism underlying the sensitization of TMZ-resistant T98G and U138 glioma cells, as found by Yang et al., was the inhibition of the Wnt signaling pathway and the downregulation of O6-methylguanine-DNA methyltransferase (MGMT) expression, which is a potent DNA repair enzyme that reverses damage from alkylating agents, like temozolomide. It is consistent with the observations of a 2012 study, which also reported decreased MGMT activity in RES-treated T98G cells. A decrease in the nuclear translocation of NF-κB and the increased cleavage of caspase-3 were also observed, supporting resistance reversal [141]. However, it was not evaluated whether these effects are long-term or transient. It is hypothesized that the modulation of autophagy by RES, reported to occur in glioblastoma cells, can contribute to resistance reversal as well since autophagy is a process known to support chemoresistance in certain cellular contexts [160].

Curcumin has been widely studied in multidrug-resistant (MDR) tumor models, including gliomas. Experimental evidence indicates that curcumin downregulates ATP-binding cassette (ABC) transporters such as P-glycoprotein and MRP1, thereby increasing intracellular concentrations of chemotherapeutic drugs. These effects resulted in enhanced apoptosis and reduced proliferation in resistant cancer cell lines, supporting curcumin’s role as a chemosensitizer [23]. To address curcumin’s poor solubility and rapid metabolism, several synthetic analogs have been developed and tested in resistant cancer models. Studies on EF24 and Go-Y030 demonstrated a potent inhibition of NF-κB and HIF-1α signaling, ROS modulation, and miRNA-mediated gene regulation, leading to cell-cycle arrest and apoptosis in resistant tumor cells. Additionally, ALZ003 was shown to induce ubiquitination and the degradation of the androgen receptor, significantly reducing the survival of therapy-resistant GBM cells [161]. Preclinical studies emphasized the use of nanocarriers and co-delivery systems to enhance curcumin bioavailability, improve brain penetration, and achieve sustained anti-resistant effects [23].

Enhanced anti-glioblastoma effects were also observed in studies combining curcumin with berberine. In vitro experiments using solid lipid curcumin particles demonstrated that co-treatment with berberine resulted in significantly increased DNA fragmentation, reduced cell viability, and stronger inhibition of the PI3K/Akt/mTOR pathway compared to monotherapy. These findings suggest that combination regimens may more effectively overcome resistance-related survival signaling [162]. A related, and more extensively developed, multi-component strategy is TriCurin, a liposomal formulation combining curcumin, resveratrol, and epicatechin gallate (a green-tea catechin) at a fixed molar ratio. In orthotopic GL261 mouse GBM models, liposomal TriCurin produced a stable plasma curcumin concentration of ~210 nM—sub-lethal to cultured GBM cells in isolation—yet it was sufficient to repolarize M2-like tumor-associated microglia/macrophages toward a tumoricidal M1-like phenotype and to recruit activated natural killer cells into the tumor, extending survival in treated animals [163]. This immune-microenvironment-directed mechanism is distinct from the direct cytotoxic pathways emphasized elsewhere in this review and illustrates that fixed-ratio, multi-compound co-formulation—rather than combining any single one of these three compounds with a conventional chemotherapeutic—is itself an actively developed translational strategy that a review covering all three compounds is well positioned to highlight.

Comparative mechanistic analyses indicate that berberine, curcumin, and resveratrol share overlapping and complementary molecular targets in glioma models. These natural compounds were shown to inhibit proliferation, invasion, and metastasis while modulating signaling pathways closely linked to treatment resistance, including PI3K/Akt, NF-κB, and STAT3. Their multi-targeted actions support their potential use as adjuncts to conventional therapies aimed at mitigating drug resistance [24].

### 4.4. Protective Effects of Curcumin, Resveratrol and Berberine on Healthy Brain Cells During Cancer Therapy

Conventional brain tumor treatment modalities such as chemo- and radiotherapy, however, despite aiming to eliminate malignant cells, often cause damage to the surrounding healthy tissue. Neurotoxicity manifests clinically as cognitive impairment, memory deficits, and reduced quality of life, collectively termed therapy-related neurocognitive decline. Therefore, protection of healthy neurons during cancer treatment, mainly in the context of treatment-induced oxidative and inflammatory insults, is also important. Medicinal plants and plant-derived compounds, exerting a wide range of biological effects, are good candidates to alleviate neurotoxicity through their antioxidant, anti-inflammatory, or antiapoptotic effects.

This protective, antioxidant role stands in direct tension with the pro-oxidant, radio-/chemosensitizing mechanisms described for the same compounds elsewhere in this review (e.g., curcumin- and resveratrol-induced ROS generation in Section 3.1.1, Section 3.2.1, and Section 4.2, and berberine-induced ER stress/ROS in Section 3.3). Which of the two effects predominates is not a fixed property of the compound but appears to depend on dose, timing relative to the cytotoxic therapy, and the redox state of the target cell: tumor cells, which often operate closer to a redox threshold, may be pushed into oxidative cell death by the same exposure that a healthy neuron tolerates or is protected by. This has a practical implication that is not, to our knowledge, adequately addressed in the clinical literature discussed in Section 5: if these compounds are taken concurrently with radiotherapy or with chemotherapeutic agents whose mechanism of action is itself oxidative (e.g., anthracyclines), there is a theoretical possibility that their antioxidant activity could blunt, rather than enhance, antitumor efficacy. We are not aware of clinical data directly testing this interaction for curcumin, resveratrol, or berberine in brain tumor patients, and we flag it here as a consideration for trial design rather than as an established risk.

An important limitation when interpreting the neuroprotective properties of curcumin, resveratrol, and berberine is that much of the supporting evidence originates from experimental models of neurodegeneration, ischemic injury, or other forms of neuronal stress rather than from glioma treatment settings. Although these findings suggest that the compounds may mitigate oxidative stress, inflammation, and cellular injury in normal neural tissues, they do not establish that such protection occurs during temozolomide-based chemotherapy or glioma radiotherapy. Moreover, the therapeutic implications of neuroprotection in oncology require particular caution because the molecular pathways involved in normal-tissue protection may also contribute to tumor cell survival. Antioxidant activity, modulation of stress-response pathways, and the inhibition of apoptosis could potentially reduce treatment-associated damage in normal cells but might also attenuate the cytotoxic effects of anticancer therapy in tumor cells. Conversely, selective effects on cellular metabolism, redox homeostasis, or stress-response signaling could theoretically produce differential responses in normal and malignant tissues. Whether such selectivity exists for curcumin, resveratrol, or berberine during glioma treatment remains insufficiently established. Therefore, neuroprotective effects reported in non-oncological models should be regarded as a rationale for further investigation rather than evidence of clinical benefit during glioma therapy. Future studies should evaluate normal neural cells and glioma cells in parallel under clinically relevant TMZ and radiotherapy conditions to determine whether normal-tissue protection can be achieved without compromising tumor control [152,156,164,165].

In the case of curcumin, it is mainly an anti-inflammatory activity that holds potential to be used as a protective agent in the treatment of brain and CNS tumors. Chronic, low-grade inflammation is generally a pro-tumorigenic factor in cancers [166] and can damage healthy brain cells as well; therefore, the use of some mild anti-inflammatory compounds may improve their function without worsening—poor as it is—brain tumor immunity. One of the suggested mechanisms of curcumin’s anti-inflammatory effect is the activation of KEAP1-Nrf2-ARE signaling pathway. Liao et al. examined the influence of curcumin on doxorubicin neurotoxic activity. CUR increased the level of nuclear Nrf2 and Keap1, as well as the expression of NQO-1 and HO-1, parts of the later stages of the signaling pathway. All mentioned elements were originally decreased by doxorubicin, which was correlated with its neurotoxicity [167]. Researchers also consider the upregulation of thioredoxin protein—an element of the Nrf2 pathway—and the induction of thioredoxin enzyme activity as the mechanism of CUR-mediated neuroprotection, as these agents are known for their antioxidant activity [168]. Moreover, curcumin treatment was observed to decrease CCL3 (chemokine (C-C motif) ligand 3) levels, as well as Toll-like receptor 4 (TLR4) and MyD88 (myeloid differentiation primary response 88), both key roles in the immune response, and p-NF-κB P65, p-p38 MAPK, and p-IκBα expression, which suggests the inhibition of TLR4/MyD88/MAPK/NF-κB inflammation-inducing signaling. A pro-inflammatory chemokine, CCL3, correlated with this pathway, was found to be upregulated in various neuropathological conditions, such as cerebral ischemia models, causing the progression of inflammation and ischemia. These results imply the mechanism of curcumin’s anti-inflammatory effect [169]. An additional underlying mechanism could be the downregulation of ER stress marker expression by curcumin: glucose-regulated protein 78 (GRP78) and activating transcription factor 4 (ATF4) mRNA. Curcumin decreases pro-apoptotic DNA damage-inducible transcription factor—nuclear CHOP—levels and increases Bax protein expression, which suggests an anti-apoptotic effect of curcumin treatment. A further example of the anti-inflammatory effect of curcumin was observed in the brain in the course of diabetes. The underlying mechanism was lowering hippocampal pro-inflammatory marker IFN-γ and oxidative stress marker MDA levels. The agent increases heat shock protein 70 (HSP70) levels, which was reported as neuro-protecting by preventing protein misfolding, as well as apoptosis and oxidative stress in the hippocampus and glial cells induced by NF-κB. CUR also enhances hippocampal ChAT and Bcl2 immunoreactivity, which achieves a neuroprotective and pro-survival outcome [170].

Several studies have shown that CUR can decrease cisplatin-related toxicity in the brain, which is mediated by several mechanisms. Firstly, the protective effect was ascribed to the antioxidant capacity of CUR through reducing mitochondrial lipid peroxidation and protein carbonyl content, as shown in rats [171]. Then, a 2020 study showed that curcumin promotes neurogenesis and synaptogenesis in the hippocampus, reducing the apoptosis rate by Bcl-2 upregulation and mTOR inhibition [172]. CUR in the form of nanoparticles (nanocurcumin, NC) was also found to be efficient in preventing doxorubicin-induced neurotoxicity. It was explained by alleviating oxidative stress by NC, but also, a prevention of the inhibition of monoaminooxidases and acetylcholine esterase has been observed, indicating an additional mechanism—a reduction in dopamine levels, increased by DOX treatment [173].

Moreover, curcumin’s ability to protect neurons against cell injury was suggested, which is of particular importance while considering invasive chemotherapy regimens that do not target only cancer cells. Curcumin pre-treatment, as well as post-treatment of primary cultured rat cortical neurons, decreased the number of cell injuries caused by oxygen-glucose deprivation [168]. The antioxidant and anti-inflammatory actions of curcumin, such as lowering glial activation, have also been linked to the alleviation of short-term and long-term memory impairment caused by doxorubicin treatment. This was examined with the novel object recognition test performed on mice treated with doxorubicin. Mice after curcumin administration had longer interaction time with novel objects at 3 h and 24 h, compared to mice with doxorubicin-only treatment. CUR was found to mitigate cognitive dysfunction by its anti-inflammatory activity as well. Changlek et al. examined the influence of curcumin on inflammation caused by lead. Mice treated with the agent did not experience a significant increase in latency time during the water maze swimming task. It also lowered depressive-like behavior, which was proven by decreased immobility time in the forced swimming test in mice. The antioxidant and anti-inflammatory character of curcumin was suggested, as it was found to bind with AChE receptors, IKK, COX-2, P38, JNK, TNF-α, and ERK and consequently reduce cholinergic alterations. The plant compound was also observed to downregulate the expression of inflammatory markers, such as TNF-α, COX-2, and iNOS [173,174].

Resveratrol has been reported to prevent chemotherapy-induced cognitive impairment in mice exposed to three-agent treatment: docetaxel, adriamycin (DOX), and cyclophosphamide (DAC regimen). A dose of 100 mg/kg was sufficient to lower levels of pro-inflammatory IL-6 and TNF-α with a concomitant increase in anti-inflammatory IL-4 and IL-10 in serum samples and brain tissues of the treated group. Mechanistically, these effects were mediated by the modulation of the PPARγ/NF-κB pathway. Moreover, RES administration prevented a decrease in neuroplasticity biomarkers, brain-derived neurotrophic factor (BDNF), tropomyosin receptor kinase B (TrkB), and calmodulin-dependent protein kinase II (CaMKII), usually observed upon DAC treatment [175]. The neuroprotective effects of RES against radiation damage were also described. In a 2020 animal model study, rats that received RES prior to radiation exposure presented a lowered oxidative stress marker concentration, increased activity of antioxidant enzymes such as glutathione peroxidase or catalase, and increased Bcl-2, confirming the potency and pleiotropy of this compound in scavenging ROS [176]. Moreover, resveratrol has been shown to protect neurons in ischemic brain injury.

Berberine exhibits neuroprotective effects as well. It has been established in similar studies to those discussed above in rats and mice, and the main chemotherapeutic used to induce neurotoxicity was DOX, with several reports evaluating ischemia-induced neural cell damage. One recent study (2024) assessed the ability of BBR, together with coenzyme Q10 or alone, in ameliorating neurotoxicity caused by a chemotherapy regimen based on cyclophosphamide, adriamycin, and 5-fluorouracil (CAF). Both compounds were shown to decrease damage to neurons by reducing oxidative stress and apoptosis markers, along with an anti-inflammatory effect by inhibiting NF-κB nuclear translocation and subsequent expression of NLRP3 and IL-1β, and through an increase in Nrf2 levels [177]. Two 2021 studies report consistent BBR protection of neurons from oxidative damage, otherwise caused by DOX treatment, by reducing expression of pro-inflammatory mediators, apoptosis-related factors, and through the antioxidant effect of BBR [178,179]. Although both the CAF regimen and DOX-only treatment are routinely used in breast cancer therapy and not brain tumors, these findings indicate that there is a rationale to further test BBR, along with CUR and RES, as neuroprotective agents during TMZ treatment.

Table 1, Table 2 and Table 3 summarize representative preclinical studies discussed in Section 3 and Section 4 for curcumin, resveratrol, and berberine, respectively; Table 4 summarizes the clinical and early translational studies discussed in Section 5. These tables compile the studies discussed in the body text of this review and are not the product of an independent systematic search.

## 5. Clinical Trials

While extensive preclinical evidence supports the anticancer potential of resveratrol, curcumin, and berberine, clinical evidence remains scarce, particularly for gliomas. Many candidates fail to advance beyond preclinical investigation, and those that do often yield modest or indirect clinical benefits. This gap reflects the unique challenges posed by central nervous system malignancies in both regulatory and biological settings, including poor systemic bioavailability, limited brain penetration, and variability in compound formulation. This section summarizes the current landscape of clinical trials assessing these compounds in brain cancer (Table 4).

### 5.1. Pharmacokinetics, Safety, and Non-CNS Evidence for Curcumin

Although curcumin has shown potential in vitro to attenuate glioblastoma malignancy through various mechanisms, its therapeutic application remains limited by its poor bioavailability, which is influenced by its large crystalline structure, low solubility, and extensive metabolism in vivo [25]. To date, no definitive clinical efficacy of CUR in brain and CNS cancers has been demonstrated. Although preclinical studies in animals have shown that high doses of CUR (e.g., 1560 mg/kg for 26 days and 180 mg/kg for 14 days) did not result in significant adverse effects, suggesting that curcumin might be safe for prolonged use at higher doses [26,81], the translation of these to clinical application in humans requires careful consideration, as the efficacy and safety of a compound like curcumin can vary significantly between laboratory models and humans. Preclinical studies often use higher doses due to various factors, including differences in metabolism rates and body sizes between animals and humans, as well as the need to achieve observable effects under experimental conditions. Therefore, it may be challenging to determine the specific clinical anticancer dose of CUR based on preclinical data alone [26].

Curcumin per se, at concentrations that fall within the physiological range, does not demonstrate intrinsic cytotoxic activity, suggesting that achievable concentrations are far below those shown to have anticancer activity in preclinical models [156]. Lao et al. in 2006 performed a dose escalation study to assess the maximum tolerated dose and safety of a single oral administration of standardized curcumin powder in a certain amount. Despite good tolerance, the compound was not detected in the serum of individuals who received doses ranging from 500 to 8000 mg. Low levels of curcumin were only detected in two subjects who received 10,000 or 12,000 mg [181].

One promising approach to overcome these limitations is local delivery of curcumin to the brain via nanoparticles, which has significantly increased its delivery to targeted brain regions [190]. In a study evaluating the bioavailability and metabolic impact of micellar curcuminoids, thirteen glioma patients received 70 mg of these orally, three times a day, for four days before surgery. Tumor and serum analyses revealed an average curcumin concentration of 56 pg/mg in glioma tissue (9–151 pg/mg) and 253 ng/mL in serum (129–364 ng/mL). Notably, changes in tumor energy metabolism were observed, such as a reduction in the phosphocreatine-to-inorganic phosphate ratio and an increase in intratumoral pH. Although the statistical significance was not reached (*p* = 0.08), the results suggest that oral micellar curcuminoids can facilitate intratumoral accumulation and may influence metabolic processes in gliomas, with the need for some improvement, e.g., in formulation [182]. Importantly, an active trial directly addresses the antitumor-efficacy gap identified above: liposomal curcumin (LC), an intravenous formulation reported to achieve plasma curcumin levels roughly 1000-fold higher than oral administration, is being evaluated in combination with standard radiotherapy and concomitant/adjuvant temozolomide in newly diagnosed high-grade glioma patients in an open-label Phase Ib-IIa dose escalation study at Johns Hopkins/NCI (NCT05768919) [183]. Preceding studies in healthy volunteers and cancer patients found LC tolerable at doses up to 300 mg/m^2^, with one case of hemolytic anemia reported in a patient also taking other hemolysis-associated drugs.

Another clinical investigation examined the use of high-dose oral renin-angiotensin system (RAS) modulators in recurrent glioblastoma patients who had already completed standard therapy. This regimen, which included turmeric/piperine tablets, propranolol, aliskiren, cilazapril, celecoxib, aspirin, and metformin, resulted in a median survival increase of over 10 weeks. Adverse events were minimal, with one patient experiencing mild dyspepsia potentially related to curcumin, which resolved without discontinuation. Survival findings should not be attributed to curcumin alone, but these findings support the further investigation of this compound in different combinations [184].

In the case of radiation-induced oral mucositis (RIOM), a frequent complication of radiotherapy for head and neck cancers, a 0.1% curcumin mouthwash was shown to delay the onset and alleviate symptom severity. Although no mouthwash has fully prevented RIOM, curcumin’s anti-inflammatory properties may significantly contribute to symptomatic relief, improving patient comfort during treatment [185].

### 5.2. Pharmacokinetics and Direct Antitumor Evidence for Resveratrol

Reviews of the clinical landscape confirm that, despite extensive preclinical in vitro and in vivo research, clinical data about resveratrol is limited due to challenges such as low bioavailability and poor brain penetration. For example, a small study involving six volunteers showed that oral administration of 25 mg RES resulted in ≥70% absorption; however, it was mainly present in plasma as metabolites, with only trace amounts of the unchanged compound detected [186]. A separate study involving 10 persons tested doses of RES ranging from 0.5 to 5 g, revealing that high doses do not reach the concentrations necessary for cancer chemoprevention (5 uM). The maximum concentration achieved after the highest dose was 2.4 uM after 1.5 h. However, elevated systemic levels of conjugated RES metabolites suggest that their potential chemopreventive properties warrant further investigation [187]. The main obstacles preventing RES from being used as a cancer therapy include its bioavailability and associated adverse events. This compound has shown varying degrees of efficiency across different cancer types. For instance, it appears to downregulate the expression of specific genes associated with breast cancer through epigenetic mechanisms, while in contrast, it has been linked to serious adverse effects in patients with multiple myeloma [191]. Moreover, oxidative enzymes, air oxidation, light exposure, and high temperatures can induce trans-to-cis isomerization, further diminishing its effectiveness [192].

Although several trials, including RES, of treatment regimens have been conducted on various types of cancers, the results were often inconclusive or ambiguous [191]. However, one very recent small pilot study (December 2025) suggested that administration of RES together with copper (R-Cu) induces the formation of oxygen radicals targeting cell-free chromatin particles released from glioblastoma cells, leading to their eradication and attenuating the aggressiveness of cancer. The low number of participants—10 patients in the treated group and 10 constituting a control group—indicates the preliminary character of this study, requiring validation in a larger cohort. Also, only biological features, such as Ki-67 expression, immune checkpoints, and stem cell biomarkers, have been assessed, while typical clinical parameters like overall survival were not evaluated therein [188].

### 5.3. Berberine: Absence of Direct Clinical Evidence in Brain Tumors

As of the current clinical research landscape, there are no completed clinical trials specifically testing berberine as an isolated plant compound in the treatment of brain or central nervous system tumors in humans that have been published. Clinical research involving berberine has so far largely remained at the preclinical stage or focused on non-CNS indications like diabetes or hyperlipidemia [189]. To date, controlled oncology trials with the use of this compound are lacking, and significant clinical evidence for berberine’s anticancer efficacy is absent.

A major challenge in implementing natural compounds is their pharmacokinetic and pharmacodynamic properties. Therefore, overcoming limitations such as bioavailability, selectivity, and permeability through the blood–brain barrier is crucial. Improving these characteristics would enhance effectiveness and achieve the desired therapeutic outcomes in various brain tumors. Clinical studies that take these factors into account are essential for this purpose [27].

In summary, the clinical investigation of these three compounds in brain and central nervous system tumors remains extremely limited. Curcumin has been evaluated in a small number of human studies, including pharmacokinetic and early-phase trials, yet no definitive evidence of clinical efficacy has been established, largely due to poor bioavailability and insufficient intratumoral exposure. Resveratrol and berberine have not progressed beyond preclinical evaluation in brain tumor patients, despite extensive preclinical data demonstrating anti-proliferative and pro-apoptotic effects in glioma models. The absence of clinical evidence for these compounds underscores the substantial translational challenges posed by unfavorable pharmacokinetics, blood–brain barrier limitations, and dosing constraints rather than a lack of mechanistic rationale. Consequently, future translational development should prioritize optimized formulations, structural analogs, and rational combination strategies with standard therapies to determine whether the promising biological activities of these natural compounds can be harnessed in a clinically meaningful manner. Importantly, the scarcity of clinical trials should not be interpreted as evidence against therapeutic potential but rather reflects the broader challenges of translating pleiotropic natural compounds into CNS-active anticancer agents.

## 6. Conclusions

Natural plant-derived compounds such as curcumin, resveratrol, and berberine represent promising multi-target agents in the treatment of gliomas. Across preclinical models, all three compounds demonstrate the ability to modulate key oncogenic pathways, including PI3K/Akt/mTOR, AMPK, MAPK, NF-κB, and Wnt/β-catenin, leading to reduced proliferation, enhanced apoptosis, inhibition of angiogenesis, and decreased invasiveness of tumor cells. Notably, these compounds also show potential to sensitize glioma cells to standard therapies, including temozolomide and radiotherapy, and in some cases mitigate treatment-induced toxicity through their antioxidant and anti-inflammatory properties.

Despite consistent preclinical mechanistic evidence, clinical translation remains very limited. Major challenges include poor bioavailability, rapid metabolism, and restricted penetration across the blood–brain barrier. Emerging nanoformulations—such as lipid nanoparticles, exosome-based carriers, and polymeric micelles—have demonstrated the ability to enhance CNS delivery and therapeutic potency, indicating that drug-delivery innovations will be essential for future clinical progress.

Overall, curcumin, resveratrol, and berberine are biologically active, multi-target compounds that warrant continued preclinical and early translational investigation as potential complements to existing therapeutic strategies for gliomas. However, rigorous in vivo studies and well-designed clinical trials are required to validate their safety, optimize dosing strategies, and confirm therapeutic efficacy in patients. Overcoming challenges related to bioavailability and delivery will be critical for harnessing the full clinical value of these natural compounds in neuro-oncology.

It must be stressed, however, that clinical efficacy in brain tumor patients remains unproven for all three compounds: as detailed in Section 5, direct clinical evidence is limited to a small number of pharmacokinetic, supportive care, and early-phase studies, several of which do not test antitumor efficacy at all, and none currently support a treatment recommendation. These compounds are not alternatives to standard neuro-oncological therapy (surgery, radiotherapy, temozolomide, and, where indicated, tumor-treating fields), and patients and clinicians should not interpret the preclinical findings summarized here as evidence of clinical benefit outside a formal trial setting.

## Figures and Tables

**Table 1:** Summary of preclinical curcumin studies discussed in this review. Upward arrow represents increase and downward arrow represents decrease or reduction.

Model	Experimental Setting	Dose/ Concentration	Administration Route	Molecular Target(s)	Reported Outcome	Limitation	Ref.
nude mice intracranially implanted with U87 cells	In vivo	60 mg/kg	intraperitoneal injection	SHH/GLI1, SMO, Cyclin D1/Bcl-2/Foxm1	↓ tumor volume, downregulated GLI1 expression, better survival	limited number of tested animals; only one glioma cell line implanted and tested	[70]
A172, KNS60, U251MG(KO)	in vitro	0, 20, 40, 60, 80 and 100 µM	addition to culture medium	CCNE1, CDK2, E2F1, PTEN	G1 arrest ↑ PTEN Telomere shortening: ↓ hTERT mRNA	in vitro only	[77]
U251	In vitro	10, 20, 30, 40, 50, and 100 μM	addition to culture medium	ENO1 (glycolysis)	↓ glycolysis, ↓ proliferation/invasion	in vitro only; only one glioma cell line used	[73]
U251, U87	in vitro	0, 10, 20, 30, and 40 μM	addition to culture medium	p-Akt/mTOR, PTEN, p53	↓ p-Akt/mTOR, ↑ PTEN/p53, ↓ tumor volume (in vivo arm)	limited diversity of the cell lines tested	[68]
female nude mice subcutaneously implanted with U87 cells	in vivo	60 mg/kg	intraperitoneal injection	p-Akt/mTOR, PTEN, p53	↓ tumor volume, weight	subcutaneous GBM xenograft; only one glioma cell line implanted and tested	
U251, SNB19	in vitro	0, 10, 15, 20, 25 and 30 μM	addition to culture medium	Skp2, pAkt, p57	↓ Skp2/pAkt, ↑ p57	in vitro only	[180]
U138MG, U87, U373, C6	in vitro	0, 7.5, 15, 30, 50 μM	addition to culture medium	PI3K/Akt, NF-κB, Bcl-xL	pro-apoptotic, impaired mitochondrial function	limited diversity of the cell lines tested	[71]
Wistar rats intracranially implanted with C6 cells	in vivo	50 mg/kg/day	intraperitoneal injection	PI3K/Akt, NF-κB, Bcl-xL	↓ tumor volume and size	only one glioma cell line implanted and tested	
U87, U251 and U235 and Patient-derived GSCs	in vitro	0, 10, 20, 30, 40, 50, 60, 70 μM	addition to culture medium	ERK/p38/JNK, STAT3, survivin/IAP1/IAP2	↓ proliferation, sphere/colony formation	in vitro only	[72]
U373	in vitro	0, 2.5, 5, 10, 15, 20 µg/mL	addition to culture medium	MMP gene expression hTERT	↓ invasion ↓ MMP-2, -14, -16 and -17 necrosis + apoptosis, telomere shortening	only one glioma cell line used	[74]
LN229, U87MG	in vitro	8, 16, 24 or 32 μmol/L	addition to culture medium	MMP-2/-9/-14/-15/-16, ERK1/2	↓ invasion, blocked NE-enhanced proliferation	in vitro only; limited diversity of the cell lines tested;	[75]
female nude mice subcutaneously implanted with LN299 cells	In vivo	60 mg/kg	intraperitoneal injection	Tumor growth induced by psychological stress	↓ tumor volume ↓ effect of psychological stress: ↓ expression of MMP-2,MMP-9 and CD147	subcutaneous GBM xenograft; only one glioma cell line implanted and tested	
A1207, SNB19	in vitro	0, 10, 15, 20 and 25 μM	addition to culture medium	NEDD4/PTEN/PI3K-AKT	↓ migration, invasion, proliferation	in vitro only	[75]
1321N1 GBM	in vitro	0.5, 1, 1.5, 2, 2.5, 3, and 6 μM	addition of curcumin nanoparticles to culture medium	DNMT3A, SUZ12, HOTAIR, MeCP2	↓epigenetic markers, ↓invasion/migration	only one glioma cell line used	[76]
U251	in vitro	0, 20, 30, 40, 50 μM	addition to culture medium	DAPK1, STAT3/NF-κB	G2/M arrest	in vitro only; only one glioma cell line used	[78]
U87 and T98G	in vitro	10, 20 μM (U87 cell line) 20, 40 μM (T98G cell line)	addition to culture medium	SHH/GLI1, SMO, Cyclin D1/Bcl-2/Foxm1	G2/M arrest, ↓ stemness	limited diversity of the cell lines tested	[70]
U-87, RBE4	in vitro	0–30 μM	addition to culture medium	CD31, CD105	↓ cell migration, ↓ proliferation	only one human glioma cell line used	[81]
U-87-derived xenografts, athymic mice (intracranially and subcutaneously implanted)	in vivo	0, 30, 60, 120 mg/kg/day	intraperitoneal injection	CD31, CD105	anti-angiogenic (↓ microvessel markers)	only one human glioma cell line implanted	
GL261	in vitro	0–25 μM	addition of free Curcumin or curcumin/MPEG-PLA drug-loaded nanoparticles or curcumin/Fa-PEG-PLA to culture medium	CD31, MMP-9	induced apoptosis	only one glioma cell line used	[82]
C57 mice intracranially implanted with GL261 cells	in vivo	50 mg/kg	intravenous injection	CD31, MMP-9	↓ new microvessels	only one glioma cell line implanted and tested	
C6, U251MG and U87MG	in vitro	0, 7.5, 15, 22.5, 30 μM CUR (+0, 50, 100, 150, 200, 250, 300, 350, 400)	addition to culture medium	STAT3, NF-κB, PI3K/AKT, ERK1/2 (autophagy)	greater cytotoxic effect with TMZ; harmless to astrocytes	limited diversity of the cell lines tested	[133]
male Wistar rats intracranially implanted with C6 cells	in vivo	50 mg/kg (+10 mg/kg TMZ)	intraperitoneal injection	STAT3, NF-κB, PI3K/AKT, ERK1/2 (autophagy)	ERK1/2 activation ↓ STAT3 phosphorylation and its DNA-binding	long-term survival not evaluated; only one glioma cell line implanted and tested	
U87MG	in vitro	0, 20, 50, 100 µM CUR (+100, 300, 500 µM TMZ)	addition to culture medium	miR-146a, NF-κB	TMZ sensitization	in vitro only; only one glioma cell line used	[134]
mouse models intracranially implanted with LN-229, U251 cells	in vivo	60 mg/kg CUR (+18 Gy RT)	intraperitoneal injection	Hedgehog signaling pathway vimentin/E-cadherin (EMT markers)	↓ tumor volume in LN-229, not U251	cell-line-dependent effect; negative arm reported	[155]
U251, LN229	in vitro	20 µM	addition to culture medium	Hedgehog signaling pathway vimentin/E-cadherin (EMT markers)	↓ hedgehog signaling pathway	Limited diversity of the cell lines tested;	
U251	in vitro	5–100 µM	addition to culture medium	radiosensitization	no effect on survival with single/fractionated RT	in vitro only; only one glioma cell line used	[156]
adult C57BL/6 male mice intracranially implanted with GL261 cells	in vivo	1.28 mM liposomal TriCurin 5 mM Curcumin liposome	intraperitoneal injection	TAM/microglia polarization (M2→M1), NK recruitment	TAM repolarization, tumor elimination reported	combination formulation; contribution of each single agent not isolated; only one glioma cell line implanted and tested	[163]

**Table 2:** Summary of preclinical resveratrol studies discussed in this review. Upward arrow represents increase and downward arrow represents decrease or reduction.

Model	Experimental Setting	Dose/Concentration	Administration Route	Molecular Target(s)	Reported Outcome	Limitation	Ref.
U251	in vitro	0, 1, 10, 25, 50 and 100 µM	addition to culture medium	Akt phosphorylation	↓ p-Akt, blocked signal transduction	only one glioma cell line used	[96]
Patient-derived GSCs, U87	in vitro	25, 50, 100 µM	addition to culture medium	Akt, p53	↓ GBM/GSC growth and infiltration	only one glioma cell line used	[106]
female BALB/c nude mice intracranially implanted with U87 cells	in vivo	25 mg/kg/day 50 mg/kg/day	administration ad libitum in the water supply; oral gavage; intracranial injection; intravenous injection	tumor volume	↓ tumor volume ~40% overall growth inhibition across routes	route-dependent efficacy not fully resolved; only one glioma cell line implanted and tested	
A172, T98G GBM	in vitro	0, 5, 50, 100 µM	addition to culture medium	Notch-1, p53	apoptosis	in vitro only	[98]
7 patient-derived glioma lines (GBM2, GBM7, G144, G179, G166, GliNS2, GBM04)	in vitro	10, 50, 100, 200 μM	addition to culture medium	Wnt signaling, Snail/Twist (EMT)	highly variable response across lines; ↓ motility ↓ Wnt signaling, ↓ Snail/Twist (EMT)	high inter-line heterogeneity acknowledged by original authors	[101]
Patient-derived GSC cultures	in vitro	50 μM	addition to culture medium	Nanog (proteasomal degradation), p53	↓ self-renewal, ↓ sphere formation, loss of stemness	only one RES concentration used	[103]
male BALB/cAJcl-nu/nu mice intracranially implanted with GSCs	in vivo	Resveratrol-treated GSCs (1 × 10^4^)	intracranial injection	differentiation of glioma stem cells	reduced tumor growth Longer survival period	RES not administered in vivo—implanted resveratrol-treated cells	
U-87MG	in vitro	0–160 μg/mL	addition to culture medium	hTERT catalytic subunit	↓ proliferation, cell division arrest	only one glioma cell line used needs in vivo confirmation (noted by original authors)	[104]
rat orthotopic glioblastoma model with implanted Rat glioblastoma RG2 cell line	in vivo	300 μM (final concentration in CSF = 50 μM)	lumbar puncture, every 3 days	STAT3, apoptosis	↑survival vs. resection alone; tumor not fully eliminated	incomplete tumor control; non-human cell lines used	[107]
Orthotopic rat GBM model with RG2 cells	in vivo	300 μM	lumbar puncture	STAT3, LC3, Beclin-1	↓ tumor size, ↑ survival; incomplete suppression	incomplete tumor control; non-human cell lines used; one RES concentration used	[110]
U-87MG	in vitro	0–400 μM	addition to culture medium	apoptosis, intracellular uptake	↑ uptake and apoptosis vs. free RES	only one glioma cell line used; carrier-specific	[111]
Female athymic NCr-nu/nu nude mice subcutaneously implanted with U87 cells	in vivo	10 mg/kg	intravenous injection	tumor growth; survival	↓ tumor growth, ↑ survival	no direct assessment of BBB penetration; subcutaneous GBM xenograft; only one glioma cell line implanted and tested	
The rat C6 glioblastoma cell line	in vitro	20 μM or 100 μM	addition to culture medium	JNK signaling pathway; improving Res administration	↓ proliferating cells, longer-lasting effect vs. control	only one glioma cell line used; non-human cell line used	[112]
male BALB/c nude mice subcutaneously implanted with C6 cells	in vivo	30 mg/kg	intraperitoneal injection	JNK signaling pathway	↑ JNK signaling, ↑ proapoptosis gene expression, ↑ apoptosis	subcutaneous GBM xenograft; only one glioma cell line implanted and tested	
SHG44 human GBM	in vitro	10 μM (+100 μM TMZ)	addition to culture medium	ROS, AMPK, Bcl-2, Ki67, GFAP	pro-apoptotic effect (greater combination effect with TMZ), G2/M arrest	only one glioma cell line used	[139]
orthotopic SHG44-implanted female BALB/cA nude mice	in vivo	40 mg/kg/day (+68 mg/kg × 3 day TMZ)	oral administration	tumor growth	↓ tumor growth	limited established in vivo targets; only one glioma cell line implanted and tested	
Rat RG-2; human LN-18, LN-428	in vitro	25, 50, 75, 100 µM RES (+250, 500, 750, 1000 µM TMZ)	addition to culture medium	MGMT, STAT3/Bcl-2/survivin	↑ apoptosis G1 or S Arrest ↓ STAT3 ↓ Survival Factors line-dependent response (LN-18 > LN-428)	very high concentrations required for human lines; in vitro only; limited diversity of the cell lines tested	[140]
T98G, A172, U87MG and SHG44	in vitro	100, 200, 400 or 800 µM RES (+50, 100, 200 or 400 µM TMZ)	addition to culture medium	MGMT	↑ apoptosis ↓ MGMT ↓ XIAP and survivin (greater combination effect with TMZ)	in vitro only; durability of effect not evaluated	[141]
U251, T98G, U138, A172, LN229	in vitro	0, 2, 4, 8, 16, and 32 μM RES (+0, 10, 25, 50, 100, 200, 400 and 800 μM TMZ)	addition to culture medium	Wnt signaling pathway	↑ apoptosis ↓ Wnt signaling pathway ↓ MGMT	in vitro only	[142]
Patient-derived GICs	in vitro	0, 20, 40, 100, 200, 400 µM RES (+100, 200, 400, 800, 1200 μM TMZ)	addition to culture medium	STAT3, DNA double-strand breaks	↑ TMZ-induced apoptosis, ↑ differentiation	limited diversity of the cell lines tested	[143]
GICs-implanted female NOD/SCID mice	in vivo	12.5 mg/kg RES (+68 mg/kg TMZ)	intraperitoneal injection	Differentiation	↓ tumor growth, ↑ differentiation	subcutaneous GICs xenograft	
U-87MG	in vitro	10–15 µM ±Hsp27 siRNA (25 nM)	addition to culture medium	HSF-1, Hsp27, caspase-3	apoptosis; strongest with RES + siRNA combination	in vitro only; only one glioma cell line used	[144]
bEnd.3 cells (mouse brain microvascular endothelial cells) C6 cells; Co-culture model BBB/C6 Glioma Spheroid	in vitro	0–6 μM 0–25 μM	addition to culture medium	BBB transport, apoptosis	↑ drug delivery/uptake, ↑ median survival	only one glioma cell line used; simplified BBB model used (not covering all important mechanisms);	[145]
Wistar rats intracranially implanted with C6 cells	in vivo	5 mg/kg	intravenous injection	Survival, Tumor growth	↓ tumor volume ↑ survival	only one glioma cell line implanted and tested	
SU-2 glioma stem/progenitor cells	in vitro	75 μmol/L	addition to culture medium	stemness markers, DNA damage repair	↓ colony/sphere formation, radiosensitization,	only one glioma cell line used	[147]
SU-2-implanted BALB/c nude mice	in vivo	150 mg/kg RES	intraperitoneal injection	Autophagy apoptosis	↓ tumor volume ↑ autophagy ↑ apoptosis	subcutaneous GSC xenograft; only one glioma cell line implanted and tested	
Intracranial C6-implanted SD rats	in vivo	40 mg/kg	oral administration	cell cycle, DNA damage, cytokines	↓ mortality, ↓ tumor volume, protected healthy tissue	only one glioma cell line implanted and tested	[148]

**Table 3:** Summary of preclinical berberine studies discussed in this review. Upward arrow represents increase and downward arrow represents decrease or reduction.

Model	Experimental Setting	Dose/Concentration	Administration Route	Molecular Target(s)	Reported Outcome	Limitation	Ref.
U-87MG, U-251MG	in vitro	50, 100, 150, 200 μM	addition to culture medium	PI3K/Akt/mTOR pathway	↑ DNA fragmentation, ↓ viability vs. monotherapy	limited diversity of the cell lines tested; in vitro only	[162]
C6 (rat)	in vitro	0, 50, 100, 200, 500 μM	addition to culture medium	Bcl-2, Bax Caspase-9, Caspase-3 Fas (CD95)	↓ Bcl-2, ↑ Bax, mitochondrial permeabilization ↑ apoptotic effectors ↑ death-receptor expression	only one glioma cell line used; in vitro only non-human cell line used	[119]
T98G	in vitro	0, 50, 75, 100, 150, 200 μg/mL	addition to culture medium	Bcl-2, Bax Caspase-9, PARP, Caspase-3	↓ Bcl-2, ↑ Bax ↑ apoptotic effectors	only one glioma cell line used; in vitro only	[120]
U251, U87, P3	in vitro	0, 50, 100, 200 μM	addition to culture medium	AMPK/mTOR/ULK1-pathway	↓ Bcl-2, ↑ Bax ↑ apoptotic effectors ↑ autophagy ↓ proliferation, migration, invasion	limited diversity of the cell lines tested	[121]
athymic mice subcutaneously implanted with U87 cells	in vivo	50 mg/kg/day	oral administration	autophagy	↑ autophagy ↓ tumor growth	subcutaneous GBM xenograft; only one glioma cell line implanted and tested	
T98G	in vitro	0, 50, 100, 150, 200 mg/mL	addition to culture medium	ROS-mediated apoptosis; ER stress	↑ intracellular Ca2 ↑ PERK, eIF2α phosphorylation (12 h incubation) ↓ PERK, eIF2α phosphorylation (18–24 h incubation) ↓ Bcl-2, ↑ Bax ↑ apoptotic effectors ER stress markers upregulated (GRP78, CHOP)	only one glioma cell line used; in vitro only	[122]
U87 MG	in vitro	10, 25, 100 and 250 µM	addition to culture medium	apoptosis via oxidative stress; AMPK activity	↓ proliferation, migration, invasion antioxidant enzymes insufficient to compensate ↑ ROS, TBARS, protein carbonylation	only one glioma cell line used; in vitro only	[123]
U87 U251	in vitro	0, 20, 40, 80 mg/L	addition to culture medium	TP53	↑ p53 phosphorylation (U87) ↓ p53 content (U251) Cell-cycle arrest	limited diversity of the cell lines tested;	[124]
BALB/c-nu male mice Subcutaneously implanted with U87 and U251 cells	in vivo	5 mg/kg	intraperitoneal injection	TP53	↓ proliferation ↓ tumor growth, volume, weight	subcutaneous GBM xenograft	
T98G LN18 SHG44 C6 LN229	in vitro	0, 25, 50, 100, 150 mg/L	addition to culture medium	ERK1/2	↓ ERK1/2 phosphorylation ↓ GSH and NADPH ↑ oncosis	no patient-derived cells; oncosis not directly proved	[125]
nude mice (BALB/c) subcutaneously implanted with C6 cells	in vivo	10 mg/kg	intraperitoneal injection	ERK1/2	↓ ERK1/2 phosphorylation ↓ tumor growth, volume, weight	only one glioma cell line implanted and tested	
Wistar rats intracranially implanted with C6 cells	in vivo	100 mg/kg	stomach infusion				
U-87 MG, U251 MG, U-118 MG, SHG-44	in vitro	0, 5, 15 µmol/L	addition to culture medium	EGFR–RAF–MEK–ERK signaling pathway	↓ proliferation ↓ EGFR–RAF–MEK–ERK	some mechanistic tests limited to 2 cell lines	[126]
BALB/c nude mice intracranially implanted with U87 cells	in vivo	50 and 100 mg/kg	oral administration	EGFR–RAF–MEK–ERK signaling pathway	↓ proliferation ↓ tumor growth	only one glioma cell line implanted and tested	
U87 U251	in vitro	6.25, 12.5, 25, 50, 100 and 200 μmol/L	addition to culture medium	VEGFR2/ERK pathway	↓ angiogenesis ↓ proliferation, migration, invasion	limited diversity of the cell lines tested	[128]
U87-implanted BALB/c nude mice (intracranial injection)	in vivo	50 mg/kg	oral administration	angiogenesis	lower vascular density ↓ tumor volume ↑ survival	only one glioma cell line implanted and tested	
U87 U251	in vitro	0, 50, 100 µM	addition to culture medium	EMT ERK1/2, IL-1β, IL-18	↓ migration ↑ β- and α-catenin ↓ vimentin and α-SMA	limited diversity of the cell lines tested; in vitro only	[129]

**Table 4:** Summary of clinical and early translational studies discussed in this review.

Registry No./Study	Phase	Population/Sample Size	Formulation & Dose	Primary Endpoint(s)	Status/Key Result	Ref.
Lao et al. 2006 dose escalation	I (PK/safety)	Healthy volunteers	Oral standardized curcumin powder, 500–12,000 mg single dose	Maximum tolerated dose; serum curcumin detection	Completed—undetectable in serum up to 8000 mg; trace levels only at 10,000–12,000 mg	[181]
Micellar curcuminoid pre-surgical study	Early-phase/window-of-opportunity	13 glioma patients enrolled; 10 completed	Oral micellar curcuminoids, 70 mg TID × 4 days pre-surgery	Tumor/serum curcumin concentration; tumor energy metabolism	Completed—tumor 56 pg/mg (9–151), serum 253 ng/mL (129–364); metabolic shifts observed	[182]
NCT05768919 (Schreck et al., Johns Hopkins/NCI)	Ib-IIa (dose escalation)	Newly diagnosed high-grade glioma	Liposomal curcumin (IV) + standard RT + TMZ; up to 300 mg/m^2^ tested previously	MTD/recommended Phase II dose; safety, tolerability	Open-label, active at time of writing (10 October 2026)	[183]
High-dose oral RAS-modulator multi-agent regimen	I	17 recurrent GBM patients post-standard therapy	Turmeric/piperine + propranolol + aliskiren + cilazapril + celecoxib + aspirin + metformin	Overall survival; tolerability	Completed—median survival +10 weeks; minimal adverse events (mild dyspepsia in 1 patient)	[184]
Curcumin mouthwash trial	Not specified	74 head & neck radiotherapy patients (non-CNS; supportive care)	0.1% curcumin mouthwash	Onset/severity of radiation-induced oral mucositis (RIOM)	Completed—delayed onset and reduced symptom severity; did not fully prevent RIOM	[185]
Healthy-volunteer resveratrol PK study	I (PK)	6 healthy volunteers	Oral resveratrol, 25 mg	Absorption, plasma speciation	Completed—≥70% absorbed, but mainly present as metabolites; unchanged RES only trace	[186]
Dose-ranging resveratrol PK study	I (PK)	10 healthy volunteers per dose level	Oral resveratrol, 0.5–5 g	Peak plasma concentration (Cmax)	Completed—Cmax 2.4 µM at 1.5 h after 5 g dose, below the ~5 µM chemopreventive target	[187]
Resveratrol + copper (R-Cu) pilot study	Pilot (December 2025)	10 GBM patients (treated) + 10 controls	Resveratrol + copper (R-Cu)	cfChP degradation; Ki-67, immune checkpoint and stem cell biomarkers	Completed (pilot)—biomarker changes reported; overall survival NOT assessed; small sample	[188]
Berberine—brain/CNS tumors	-	-	-	-	No registered or published clinical trials of isolated berberine for brain/CNS tumor patients	[189]

## Data Availability

No new data were created or analyzed in this study. Data sharing is not applicable to this article.

## Abbreviations

The following abbreviations are used in this manuscript:

CNS	central nervous system
GBM	glioblastoma
PCNSL	primary central nervous system lymphoma
BMs	brain metastases
BBB	blood–brain barrier
MDR	multidrug resistance
MGMT	O-6-methylguanine-DNA methyltransferase
TERT	telomerase reverse transcriptase
ECM	extracellular matrix
CUR	curcumin
PTEN	phosphate and tensin homologue
Skp2	S-phase kinase-associated protein 2
GSC	glioblastoma stem cell
MMP	matrix metalloproteinase
NE	exogenous norepinephrine
DAPK1	death-associated protein kinase 1
SHH/GLI1	Sonic Hedgehog/glioma-associated oncogene homolog 1
SMO	Smoothened
Cdc27	cell division cycle protein 27
APC/C	anaphase promoting complex/cyclosome
hTERT	Human telomerase reverse transcriptase
MMP-9	matrix metalloproteinase 9
SLCP	solid lipid curcumin particles
RES	resveratrol
SIRT1	sirtuin 1
SET7/9	lysine methyltransferase 7/9
AMPK	AMP-activated protein kinase
IκB	κB inhibitor
GSC	glioblastoma stem-like cell
GI	gastrointestinal
BBR	berberine
CD95	death receptor Fas
ULK1	Unc51-like kinase 1
PERK	PKR-like ER kinase
TBARS	thiobarbituric acid reactive substances
wtp53	wild-type p53
mutp53	mutant p53
GSH	reduced glutathione
EMT	epithelial–mesenchymal transition
TMZ	temozolomide
TF	turmeric force
GFAP	glial fibrillary acid protein
GICs	glioblastoma-initiating cells
HSF-1	heat shock factor-1
RT	radiotherapy
CSC	cancer stem cell
ABC	ATP-binding cassette
CCL3	chemokine (C-C motif) ligand 3
TLR4	toll-like receptor 4
MyD88	myeloid differentiation primary response 88
GRP78	glucose-regulated protein 78
ATF4	activating transcription factor 4
HSP70	heat shock protein 70
NC	nanocurcumin
BDNF	brain-derived neurotrophic factor
TrkB	tropomyosin receptor kinase B
CaMKII	calmodulin-dependent protein kinase II
CAF	cyclophosphamide, adriamycin, and 5-fluorouracil
PEG	polyethylene glycol
RAS	renin-angiotensin system
RIOM	radiation-induced oral mucositis
R-Cu	resveratrol with copper
